# *Mycoplasma pneumoniae* carriage in children with recurrent respiratory tract infections is associated with a less diverse and altered microbiota

**DOI:** 10.1016/j.ebiom.2023.104868

**Published:** 2023-11-10

**Authors:** Mischa H. Koenen, Ruben C.A. de Groot, Wouter A.A. de Steenhuijsen Piters, Mei Ling J.N. Chu, Kayleigh Arp, Raïza Hasrat, Ad C.J.M. de Bruijn, Silvia C. Estevão, Erhard van der Vries, Jeroen D. Langereis, Marianne Boes, Debby Bogaert, Annemarie M.C. van Rossum, Wendy W.J. Unger, Lilly M. Verhagen

**Affiliations:** a Center of Translational Immunology, UMC Utrecht, Utrecht, the Netherlands; b Laboratory of Pediatrics, Division of Pediatric Infectious Diseases and Immunology, Erasmus MC University Medical Center Rotterdam – Sophia Children's Hospital, Rotterdam, the Netherlands; c Centre for Infectious Disease Control, National Institute for Public Health and the Environment, Bilthoven, the Netherlands; d Center for Inflammation Research, Queen's Medical Research Institute, University of Edinburgh, Edinburgh, United Kingdom; e Department of Pediatric Infectious Diseases and Immunology, Wilhelmina Children's Hospital, Utrecht, the Netherlands; f Department of Research & Development, GD Animal Health, Deventer, the Netherlands; g Laboratory of Medical Immunology, Department of Laboratory Medicine, Radboud Center for Infectious Diseases, Radboud University Medical Center, Nijmegen, the Netherlands; h Division of Pediatric Infectious Diseases and Immunology, Department of Pediatrics, Erasmus MC University Medical Center Rotterdam – Sophia Children's Hospital, Rotterdam, the Netherlands; i Department of Pediatric Infectious Diseases and Immunology, Amalia Children's Hospital, Radboud University Medical Center, Nijmegen, the Netherlands

**Keywords:** *Mycoplasma pneumoniae*, Microbiota, Immunoglobulin A, *Haemophilus influenzae*, Children, Recurrent respiratory tract infections

## Abstract

**Background:**

*Mycoplasma pneumoniae* is a common cause of community-acquired pneumonia in school-aged children and can be preceded by asymptomatic carriage. However, its role in recurrent respiratory tract infections is unclear. We studied the prevalence of *M.pneumoniae* carriage in children with recurrent respiratory infections and identified associated factors.

**Methods:**

We tested *M.pneumoniae* carriage by qPCR in children with recurrent infections and their healthy family members in a cross-sectional study. Serum and mucosal total and *M.pneumoniae-*specific antibody levels were measured by ELISA and nasopharyngeal microbiota composition was characterized by 16S-rRNA sequencing.

**Findings:**

Prevalence of *M.pneumoniae* carriage was higher in children with recurrent infections (68%) than their family members without infections (47% in siblings and 27% in parents). *M.pneumoniae* carriage among family members appeared to be associated with transmission within the household, likely originating from the affected child. In logistic regression corrected for age and multiple comparisons, IgA (OR 0.16 [0.06–0.37]) and total IgG deficiency (OR 0.15 [0.02–0.74]) were less prevalent in *M.pneumoniae* carriers (n = 78) compared to non-carriers (n = 36). In multivariable analysis, the nasopharyngeal microbiota of *M.pneumoniae* carriers had lower alpha diversity (OR 0.27 [0.09–0.67]) and a higher abundance of *Haemophilus influenzae* (OR 45.01 [2.74–1608.11]) compared to non-carriers.

**Interpretation:**

*M.pneumoniae* carriage is highly prevalent in children with recurrent infections and carriers have a less diverse microbiota with an overrepresentation of disease-associated microbiota members compared to non-carriers. Given the high prevalence of *M.pneumoniae* carriage and the strong association with *H. influenzae*, we recommend appropriate antibiotic coverage of *M.pneumoniae* and *H. influenzae* in case of suspected pneumonia in children with recurrent respiratory tract infections or their family members.

**Funding:**

Wilhelmina Children’s Hospital Research Fund, ‘Christine Bader Stichting Irene KinderZiekenhuis’, Sophia Scientific Research Foundation, ESPID Fellowship funded by 10.13039/501100023280Seqirus, Hypatia Fellowship funded by 10.13039/501100006209Radboudumc and The Netherlands Organisation for Health Research and Development (10.13039/100000001ZonMW VENI grant to LM Verhagen).

**Research in context**

**Evidence before this study**

Recurrent respiratory tract infections frequently affect young children and cause considerable morbidity. Following the introduction of pneumococcal conjugate vaccines, *Mycoplasma pneumoniae* has emerged as a common bacterial cause of community-acquired pneumonia in children. *M.pneumoniae* pneumonia can be preceded by asymptomatic upper respiratory tract carriage.

**Added value of this study**

This study shows that *M.pneumoniae* carriage is highly prevalent in young children suffering from recurrent respiratory infections and is associated with a lower microbiota diversity and an overrepresentation of the disease-associated bacterium *Haemophilus influenzae* in the nasopharyngeal microbiota.

**Implications of all the available evidence**

The findings from this study shed new light on *M.pneumoniae* carriage in young children and reveal previously unrecognized associations between *M.pneumoniae* carriage and the local respiratory microbiota, contributing to the understanding of possible host defense mechanisms against *M.pneumoniae* carriage and infections.

Given the high prevalence of *M.pneumoniae* carriage in children with recurrent respiratory infections and their family members and the strong association of *M.pneumoniae* carriage with *H. influenzae* abundance, we recommend antibiotic treatment with appropriate coverage of *M.pneumoniae* and *H. influenzae* in case of suspected pneumonia in children with rRTIs or their family members.

## Introduction

Recurrent respiratory tract infections (rRTIs) affect 10–15% of young children,^1^^,^^2^ who may experience lung function loss and chronic obstructive pulmonary disease as a result.^3^ Following the introduction of pneumococcal conjugate vaccines, *Mycoplasma pneumoniae* has emerged as the most prevalent bacterial cause of community-acquired pneumonia (CAP) in hospitalized children in Western countries.^4^^,^^5^ *M.pneumoniae* is a member of the class of *Mollicutes* and like its class members, lacks a cell wall, thereby making *M.pneumoniae* inherently resistant to beta-lactam antibiotics. Besides causing pneumonia, *M.pneumoniae* infections can result in a variety of extra-pulmonary manifestations including mucocutaneous and neurological complications.^6^ Like other respiratory pathogens, *M.pneumoniae* pneumonia can be preceded by asymptomatic *M.pneumoniae* carriage in the upper respiratory tract (URT).^7^^,^^8^ *M.pneumoniae* carriage can last for several months and is in itself asymptomatic.^8^^,^^9^ *M.pneumoniae* carriage also forms a reservoir for horizontal transmission to other hosts that are in close contact, such as household members.^10^, ^11^, ^12^

Carriage of potential pathogens is influenced by various factors, including (pathogen-specific) mucosal antibodies^13^, ^14^, ^15^, ^16^ and the local respiratory microbiota,^17^ which in itself is also associated with the development of rRTIs.^18^^,^^19^ Primary antibody deficiencies are common in children suffering from rRTIs.^20^^,^^21^ In *M.pneumoniae* infections, *M.pneumoniae*-specific antibody responses play a pivotal role in clearing *M.pneumoniae* from the lungs.^13^ In B-cell deficient μMT mice, *M.pneumoniae* infections lead to chronic disease, characterized by failure to thrive and more severe pneumonia due to bacterial persistence in the lungs.^13^ Consistent with the findings obtained in mice, it has been shown that primary antibody deficiencies in humans are associated with increased frequency and severity of *M.pneumoniae* infections.^22^ However, knowledge on *M.pneumoniae* carriage in the URT of children with rRTIs, with or without antibody deficiencies, is limited. Therefore, in this study we aimed to determine what factors are associated with *M.pneumoniae* URT carriage, including antibody levels, and respiratory microbiota composition, in children with rRTIs. We hypothesized that the (relative) lack of *M.pneumoniae*-specific antibodies in the URT and an altered URT microbiota composition could contribute to *M.pneumoniae* carriage in children with rRTIs. We investigated this hypothesis by: 1) assessing the prevalence of *M.pneumoniae* carriage in the URT of children with rRTIs and their family members without rRTIs; 2) detecting the presence of *M.pneumoniae*-specific antibodies in nasopharyngeal swabs of children carrying *M.pneumoniae* compared to non-carriers; 3) examining the URT microbiota in children with rRTIs and comparing microbiota diversity and members between *M.pneumoniae* carriers and non-carriers.

## Methods

### Study population

As part of the PID-DIMER study,^23^ we enrolled children between six weeks and eight years old with rRTIs undergoing immunological screening, as well as their siblings and parents, between 01-03-2016 and 01-01-2019 in our cross-sectional study. Family members were only included if they did not suffer from rRTIs, consented to collection of both serum and a nasopharyngeal (NP) swab and were sampled only during periods when they were not experiencing an acute RTI. rRTIs were defined according to the guideline of the Dutch Section of Pediatric Infectious Diseases & Immunology,^24^ based on definitions by Gruber et al.,^25^ as: ≥11 upper RTIs per year for children up to two years, ≥8 upper RTIs per year for children aged between two and five years, ≥6 upper RTIs per year for children aged between five and eight years, or ≥2 pneumonia episodes diagnosed in one year or ≥3 pneumonia episodes diagnosed during the lifetime of the child. The choice for immunological screening was based on the physician's professional opinion after clinical consultation and physical examination. Furthermore, children with an already known IgA deficiency (<−2SD for age-appropriate reference values, see Supplementary Methods) diagnosed within the year before inclusion, were also included. Exclusion criteria for the study were: known primary immunodeficiencies requiring immunoglobulin substitution, secondary immunodeficiencies, major congenital anomalies, antibiotic use four weeks prior to inclusion and/or azithromycin prophylaxis three months prior to sampling, as this could influence *M.pneumoniae* carriage.

### Ethics

Ethical approval was obtained from the Medical Ethical Committee of the Erasmus MC (METC:NL40331.078). Legal guardians signed informed consent and the study was carried out in accordance with the Declaration of Helsinki.

### Sample and data collection

One serum sample and one NP swab were obtained from all children with rRTIs at inclusion or at the first available timepoint when there was no antibiotic use in the previous month. NP swabs were collected with standardized procedures (Supplementary Methods) and stored in RNA protect (QIAGEN, Supplementary Methods). Samples were shipped on dry ice and stored in a −80 °C freezer until further processing.

At inclusion, (caretakers of) subjects were asked to fill in a questionnaire on their medical history, underlying conditions, RTI symptoms (including fever) at time of sampling and medication use. An acute RTI was defined as two or more RTI symptoms for a minimum of two days. Furthermore, a longer questionnaire on medical history, allergies, and asthma was sent to all caretakers of children with rRTIs. Additionally, hematological and immunological results from laboratory testing conducted at inclusion were extracted from the electronic patient files.

### Immunoglobulin measurements in serum and NP swabs

Serum immunoglobulin (Ig)A, IgM, IgG, and IgG subclass levels were measured as part of routine clinical care Supplementary Material and IgE was additionally measured (Supplementary Material). An antibody deficiency was defined as immunoglobulin (isotype) levels 2SD below age-appropriate reference values as used in standard Dutch clinical care^26^^,^^27^ (Supplementary Materials and study protocol). IgA measurements to determine the presence of asymptomatic IgA deficiency were conducted in parents and siblings from whom serum was collected. *M.pneumoniae-*specific and total IgA were measured in NP swabs as previously described.^13^

### Detection of *M.* pneumoniae, viruses and *Haemophilus* via quantitative polymerase chain reaction (qPCR)

DNA was isolated from NP swabs using QIAamp DNA minikit (QIAGEN) and *M.pneumoniae* DNA was detected with in-house real-time qPCR (RT-qPCR) and *M.pneumoniae* DNA load was quantified using a plasmid dilution series as previously described,^28^ with some swabs being analyzed multiple times (Supplementary Methods). Bacterial loads were expressed as *M.pneumoniae* genome copies per mL original sample. A child was considered a *M.pneumoniae* carrier when they were positive (>100 copies/mL) one or more times. A subset of the NP samples were also analyzed by real-time qPCR to detect 12 respiratory viruses (Supplementary Table S1) according to manufacturer's instructions.^29^ We conducted a qPCR to subtype *Haemophilus* with *hypD* and *siaT* primers previously described,^30^ in all NP swabs with ≥10% relative abundance (RA) of *H. influenzae/haemolyticus* in their URT microbiota (Supplementary Methods).

### High-throughput 16S ribosomal RNA sequencing of NP samples

Bacterial DNA was isolated as previously described.^31^ Only samples with a bacterial density 0.2 pg/μl above the negative controls as measured with RT-qPCR were further analyzed. Amplicon libraries of the V4-region of the 16S ribosomal RNA (16S-rRNA) gene were generated with primer pair 515F [GTGCCAGCMGCCGCGGTAA] and 806R [GGACTACHVGGGTWTCTAAT].^32^ Amplicon pools were sequenced in five runs using the Illumina MiSeq platform. Paired-end reads were filtered and trimmed (maxEE = 2; truncLen = 180/140bp), merged, denoised, chimera filtered, and binned into amplicon sequence variants (ASVs) using DADA2 (v1.16.0).^33^ DADA2-implementation of the naïve Bayesian classifier was used for taxonomic assignment using the Silva v138v2 (August 2020) reference database.

### Downstream handling of respiratory microbiota sequencing data

Non-bacterial ASVs and potential contaminants (decontam-package v1.14.0, either-method with threshold of 0.3 and manual inspection of negative control samples per run), and samples below 10,000 reads were removed. After a presence-abundance filter (ASVs with a RA > 0.1% in ≥2 samples^34^), ASVs were clustered on 99% similarity with the IdClusters() function in the DECIPHER-package (v2.22.0, single-method, cutoff 0.01) and annotation were individually checked using BLAST (NCBI database). When multiple species were identified, all possible annotations were presented. Clustered ASVs with ≥1% RA detected in ≥10% of children, were further investigated in relation to *M.pneumoniae* carriage. Absolute abundance was determined by multiplying RA with the 16S concentration measured.

### Exploring the 16S-rRNA sequence of *M.pneumoniae* in the NP microbiota

To address the absence of *M.pneumoniae* in the 16S-rRNA sequencing dataset, we investigated our primers and probes in relation to *M.pneumoniae* sequences (n = 3) from the Silva database, as well as a *Staphylococcus aureus* and *Streptococcus pneumoniae* as positive controls. We discovered a consistent single nucleotide mismatch in all *M.pneumoniae* sequences with the forward primer (Supplementary Information), which was not observed for *S. aureus* and *S. pneumoniae*. Next, we performed 16S qPCR on three *M.pneumoniae* strains (FH, M129, and MAC) and *M.pneumoniae* positive patient samples that were spiked with *M.pneumoniae* strains using the same primers and probes, as well as the 16S RT-qPCR mentioned earlier. Lastly, to determine whether the inability to detect *M.pneumoniae* was due to the single nucleotide mismatch, we compared new primers without this mismatch to our original primers.

### Statistics

All analyses were conducted using R (v4.1.2) and R studio (v1.4.1103). All analyses were corrected for age. Baseline characteristics were compared using Chi-square/Fishers exact test and Mann–Whitney U test. Alpha diversity was measured using the Shannon index (vegan package v2.5-7) based on total ASVs before filtering and beta diversity on the Bray–Curtis dissimilarity matrix. Global microbiota compositional differences between groups were assessed using permutational multivariate analysis of variance (PERMANOVA, adonis2 function; vegan package v2.5-7). We performed hierarchical clustering of microbiota profiles based on Bray–Curtis dissimilarities, determining the optimal number of clusters with Caliński-Harabasz measure and Silhouette index. All further microbiota analyses were based on total sum scaled (relative) abundances, unless mentioned otherwise. The relationship between antibody levels and/or RA of microbiota members was assessed using Spearman correlation metrics. To identify variables associated with *M.pneumoniae* carriage, we performed univariable and multivariable logistic regression. Mucosal and serum antibody levels, as well as antibody deficiencies, associated with *M.pneumoniae* carriage were compared using logistic regression analyses corrected for age and multiple testing (Benjamini-Hochberg). Prior to multivariable analysis, missing data were imputed using the R mice package (v3.13.0, 50 iterations, pmm method). We selected the most parsimonious model based on backward elimination of variables, with age as a fixed variable, comparing the fit of subsequent models based on Akaike Information Criterion (AIC). P-values from two-sided tests below 0.05 were considered statistically significant.

### Role of funders

This work was supported by the Wilhelmina Children's Hospital Research Fund and ‘Christine Bader Stichting Irene KinderZiekenhuis’. Dr. Verhagen received an ESPID Fellowship funded by Seqirus, a Hypatia Fellowship funded by the Radboudumc, and a VENI grant from The Netherlands Organisation for Health Research and Development [grant number 09150162010077]. Ruben de Groot received a grant from the Sophia Scientific Research Foundation [grant number S18-04].

The funders had no role in the study design; in the collection, analysis and interpretation of data; in the writing of the report; or in the decision to submit the paper for publication.

## Results

**Fig. 1:**
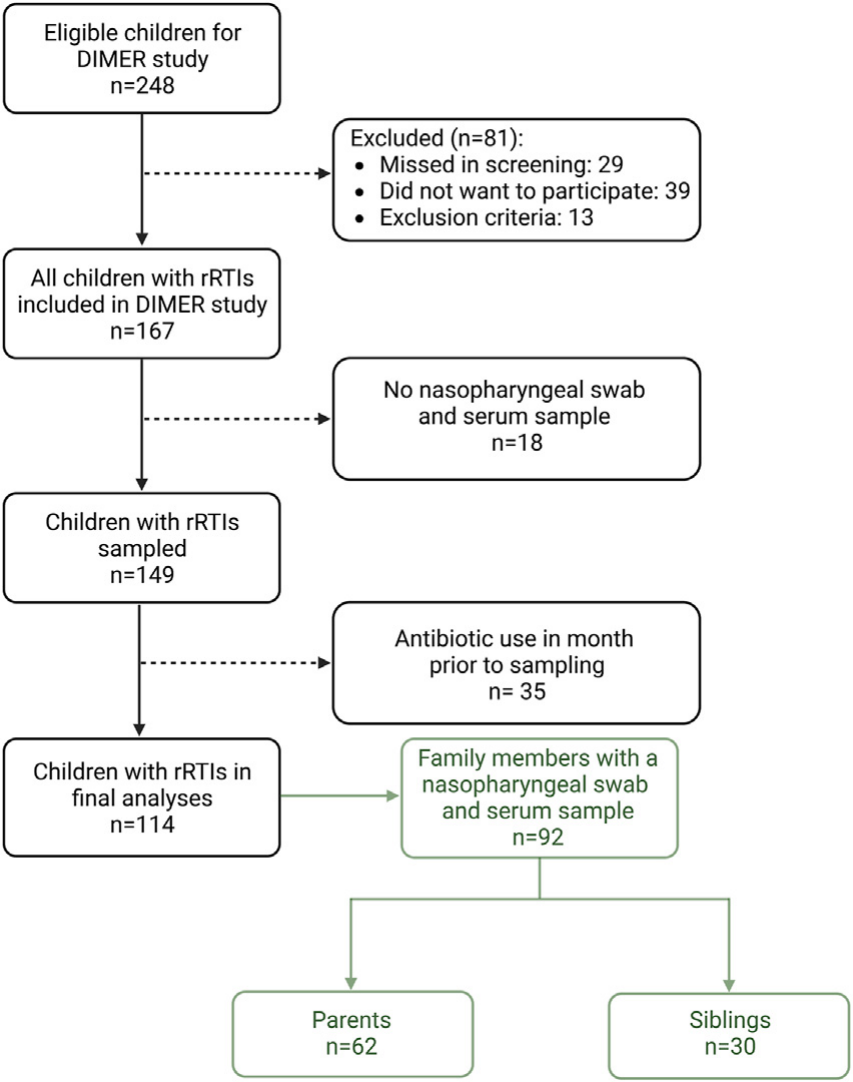
**Flowchart of children with recurrent respiratory tract infections and their family members included in the study**.

We enrolled a total of 114 children with rRTIs, 30 unaffected siblings, and 62 unaffected parents (Fig. 1). We included a higher number of family members of IgA deficient children, and we conducted IgA measurements for all family members to identify asymptomatic IgA deficiency. The mean prevalence of *M.pneumoniae* URT carriage was 53%: 68% (95% confidence interval (CI) 59–76%) for children with rRTIs, 47% (95% CI 30–64%) for siblings without rRTIs, and 27% (95% CI 18–40%) for parents without rRTIs. Notably, there was no regional *M.pneumoniae* epidemic at the time of sampling (Supplementary Figure S1A).

We examined transmission among household members (n = 92) from the 37 included households using two approaches. First, we found that individuals in households with more *M.pneumoniae*-positive members had a higher likelihood of being carriers themselves. The probability that a household member was a *M.pneumoniae* carrier was 26% with no positive household members, 36% for one, 41% for two, and 77% for three or more household members who were *M.pneumoniae* carriers (p < 0.01).

Second, we compared chances of cohabiting with at least one other *M.pneumoniae*-positive household member for carriers versus non-carriers, and found a significant difference (67% versus 18%, p < 0.01), while the total number of included family members was similar for carriers and non-carriers (4 [3–5] and 4 [3–4], respectively, p = 0.22).

*M.pneumoniae* carrier status in household members was significantly associated with the carrier status of the child with rRTIs. Among family members with *M.pneumoniae* carriage, 74% (23/31) had a *M.pneumoniae*-positive child with rRTIs, compared to 39% (24/61) of family members without *M.pneumoniae* carriage (p < 0.01). For rRTI children, when the child was *M.pneumoniae* positive, 25% [IQR 0%–38%] of family members were carriers, versus 0% [IQR 0%–29%] when the child was *M.pneumoniae* negative (p = 0.12).

Age, prophylactic antibiotic use, history of recurrent pneumonia, season of sampling, fever and/or RTI symptoms at time of sampling did not differ between *M.pneumoniae* carriers and non-carriers (Table 1, Supplementary Table S2). In children with rRTIs, none of the standard clinical hematological or immunological laboratory test results that could indicate an acute *M.pneumoniae* infection, differed between *M.pneumoniae* carriers and non-carriers. Also, we found no association between allergic predisposition or IgE levels and *M.* *pneumoniae* carriage (Table 1). In addition, we compared children with rRTIs with and without an acute RTI present at time of sampling and found a similar prevalence of *M.pneumoniae* carriage (Supplementary Table S3). Apart from C-reactive protein (CRP), which was higher in children sampled during an acute RTI, none of the clinical, hematological or immunological variables differed between the groups.

**Table 1:** Study demographics of children with recurrent respiratory tract infections stratified by *M.pneumoniae* carriage status.

	*M.pneumoniae* carriers (n = 78)	Non-carriers (n = 36)	p-value
Age in years (median [IQR^a^])	3.7 [2.2–5.4]	4.1 [2.7–5.3]	p = 0.36
Female sex assigned at birth	42% (33/78)	44% (16/36)	p = 0.99
Recurrent pneumonia	16% (12/78)	17% (6/36)	p = 1.00
Any virus detected	46% (12/26)^d^	6% (1/16)^d^	p < 0.01
Rhinovirus	35% (9/26)	0% (0/16)	
Rhinovirus and parainfluenza virus 1	4% (1/26)	0% (0/16)	
Parainfluenza virus 2	0% (0/26)	6% (1/16)	
Coronavirus OC43	4% (1/26)	0% (0/16)	
Coronavirus NL63	4% (1/26)	0% (0/16)	
Any prophylactic antibiotics prior to sampling	27% (21/77)	43% (15/35)	p = 0.16
Co-trimoxazole	23% (18/77)	34% (12/35)	
Amoxicillin	1% (1/77)	9% (3/35)	
Amoxicillin/clavulanic acid	1% (1/77)	0% (0/35)	
Trimethoprim	1% (1/77)	0% (0/35)	
Sampled in winter	22% (17/78)	14% (5/36)	p = 0.46
Fever at time of sampling	6% (8/68)	7% (2/30)	p = 1.00
Infection category at time of sampling			p = 0.94
No RTI^b^ symptoms	61% (42/69)	59% (19/32)	
≤2 RTI^b^ symptoms for ≤2 days	16% (11/69)	19% (6/32)	
>2 RTI^b^ symptoms for >2 days	23% (16/69)	22% (7/32)	
Number of times the same NP^c^ swab was analyzed per subject			p = 0.95
1 time analyzed	67% (52/78)	64% (23/36)	
2 times analyzed	23% (18/78)	25% (9/36)	
3 times analyzed	10% (8/78)	11% (4/36)	
Extra-pulmonary manifestations of *M.pneumoniae*	0% (0/78)	0% (0/36)	NA
Asthma/recurrent wheezing	49% (34/69)	55% (17/31)	p = 0.77
Allergic rhinitis	45% (29/65)	70% (21/30)	p = 0.04
Allergic dermatitis	51% (35/69)	55% (17/31)	p = 0.87
Food allergy	23% (16/69)	23% (7/31)	p = 1.00
Autoimmune disease	0% (0/78)	3% (1/36) Celiac disease (n = 1)	p = 0.32
Extra-pulmonary diseases	0% (0/78)	0% (0/36)	NA
Hemoglobulin (mmol/L, median [IQR^a^])	7.6 [7.2–8.1]	7.7 [7.5–8.0]	p = 0.37^e^
Erythrocytes (count x10^12^/L, median [IQR^a^])	4.5 [4.3–4.8]	4.6 [4.4–4.8]	p = 0.38^e^
C-reactive protein (mg/L, median [IQR^a^])	0.5 [0.5–1.0]	0.5 [0.5–1.6]	p = 0.67^e^
White blood cells (count x10^9^/L, median [IQR^a^])	10.3 [8.6–11.7]	8.2 [6.4–11.2]	p = 0.20^e^
Lymphocytes (count x10^9^/L, median [IQR^a^])	4.5 [3.4–5.7]	4.1 [2.6–4.7]	p = 0.33^e^
Monocytes (count x10^9^/L, median [IQR^a^])	0.68 [0.55–0.80]	0.65 [0.53–0.72]	p = 0.41^e^
Neutrophilic granulocytes (count x10^9^/L, median [IQR^a^])	3.7 [3.0–5.6]	3.4 [2.5–4.4]	p = 0.32^e^
Basophilic granulocytes (count x10^9^/L, median [IQR^a^])	0.06 [0.01–0.09]	0.05 [0.03–0.09]	p = 0.34^e^
Eosinophilic granulocytes (count x10^9^/L, median [IQR^a^])	0.24 [0.16–0.45]	0.20 [0.10–0.31]	p = 0.17^e^
Immunoglobulin E (kU/L, median [IQR^a^])	33 [12–154]	29 [12–82]	p = 0.13^e^

a IQR = Interquartile range.

b RTI = Respiratory tract infection.

c NP = Nasopharyngeal.

d Viral qPCR panel was only performed on a subset of children with rRTIs

e p-value from logistic regression corrected for age.

In a random subset of children with rRTIs (42/114), we measured a panel of respiratory viruses. *M.pneumoniae* carriers had a higher co-detection rate of respiratory viruses compared to non-carriers (46% and 6% respectively, p < 0.01, Table 1).

### Serum antibody levels in children with rRTIs were higher in *M.pneumoniae* carriers

**Fig. 2:**
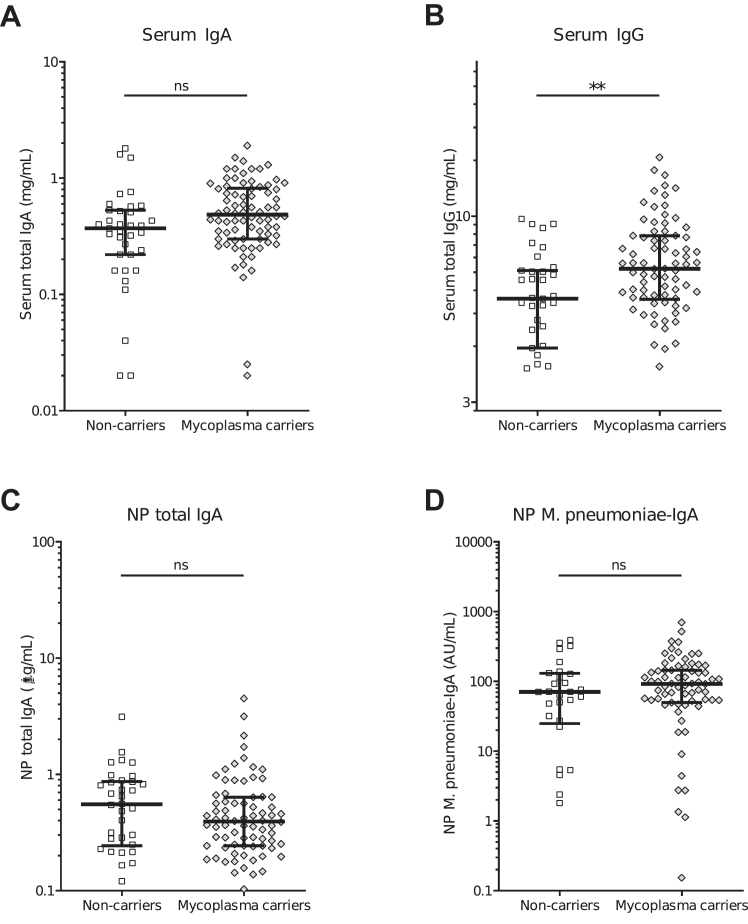
**Antibody levels in children with recurrent respiratory tract infections.** (A–D) Antibody levels in children with rRTIs. (A) Serum total IgA levels in *M.pneumoniae* carriers and non-carriers. (B) Serum total IgG levels in *M.pneumoniae* carriers and non-carriers. (C) Nasopharyngeal swab total IgA levels in *M.pneumoniae* carriers and non-carriers. (D) Nasopharyngeal swab *M.pneumoniae-*specific IgA levels in *M.pneumoniae* carriers and non-carriers. Lines represent medians and error bars show interquartile ranges. ∗p < 0.05, ∗∗p < 0.01 (logistic regression analysis corrected for age and multiple comparison).

*M.pneumoniae* carriers had a lower prevalence of IgA deficiency (OR 0.16 [95% CI 0.06–0.37], p < 0.001, adjusted for age and multiple comparisons) and total IgG deficiency (OR 0.15 [95% CI 0.02–0.74], p = 0.03, adjusted for age and multiple comparisons) compared to non-carriers with rRTIs. Additionally, *M.pneumoniae* carriers had higher levels of total serum IgG (OR 1.52 [95% CI 1.18–2.02], p < 0.01, adjusted for age and multiple comparisons) and IgG1 subclass (OR 1.75 [95% CI 1.24–2.59], p < 0.01, adjusted for age and multiple comparisons), however, there were no differences in absolute serum IgA and IgM levels (Fig. 2A and B, Supplementary Figure S1B and C). As we mostly included family members of IgA deficient individuals, IgA was measured in all family members to identify asymptomatic IgA deficiency. While IgA deficiency was found in 13% (4/30) of siblings and 10% (6/62) of parents, it did not differ between *M.pneumoniae* carrier and non-carrier family members (Supplementary Table S2A and B).

Serum IgA and total IgG levels were correlated in children with rRTIs (Spearman correlation 0.52, p < 0.001). There was no correlation between serum and NP swab IgA levels (Supplementary Figure S1D). Also, NP swab total IgA and *M.pneumoniae-*specific IgA levels did not differ between *M.pneumoniae* carriers and non-carriers (Fig. 2C and D).

### Microbiota composition differed between children with and without *M.pneumoniae* carriage

NP swab sequencing was successful in 99 children with a median of 41,917 reads (range 12,034–67,949) per sample. Prior to filtering, 4604 ASVs were detected. We removed 571 possible contaminants and 431 non-bacterial ASVs. After filtering out ultra-rare ASVs, 99 ASVs remained for downstream analyses. *M.pneumoniae* was not detected by 16S-rRNA sequencing. Further examination of our pipeline revealed that the V4 16S gene from three isolated *M.pneumoniae* strains could be detected when concentrations were very high, but the signal was rapidly lost when concentrations decreased, regardless of the V4 primers used (Supplementary Figure S2A and B).

**Fig. 3:**
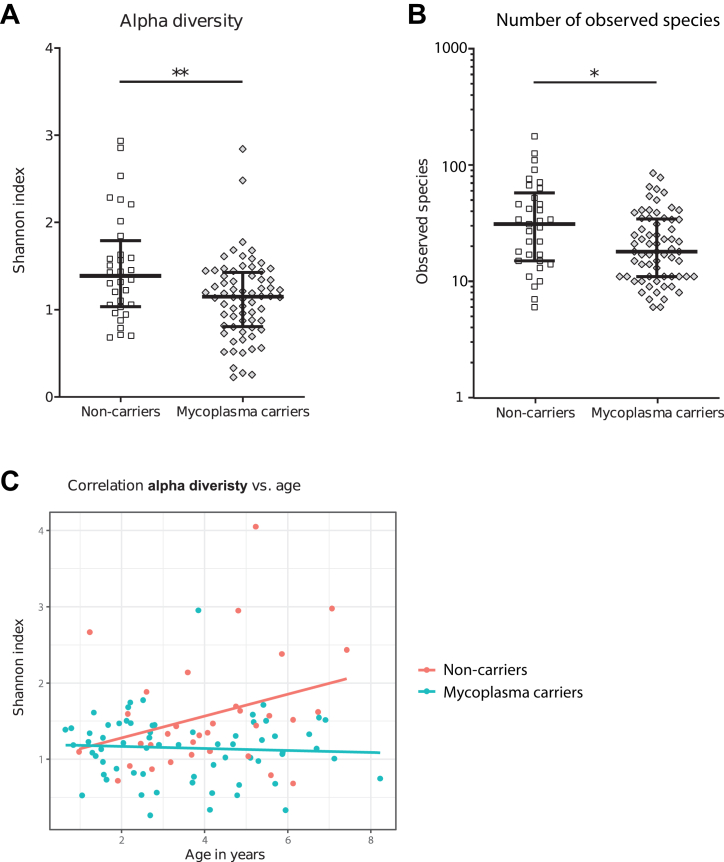
**Nasopharyngeal microbiota alpha diversity in *M.pneumoniae* carriers versusnon-carriers.** Distribution of alpha diversity (A, Shannon) and richness (B, number of observed species) of the respiratory microbiota in both *M.pneumoniae* carriers and non-carriers with rRTIs. (C) Spearman correlation of alpha diversity with age for *M.pneumoniae* carriers (Spearman correlation coefficient −0.064, p = 0.61) and non-carriers (correlation coefficient 0.318, p = 0.07). (A–B) Lines represent medians and error bars show interquartile ranges. ∗∗p < 0.01 (logistic regression analysis corrected for age).

**Fig. 4:**
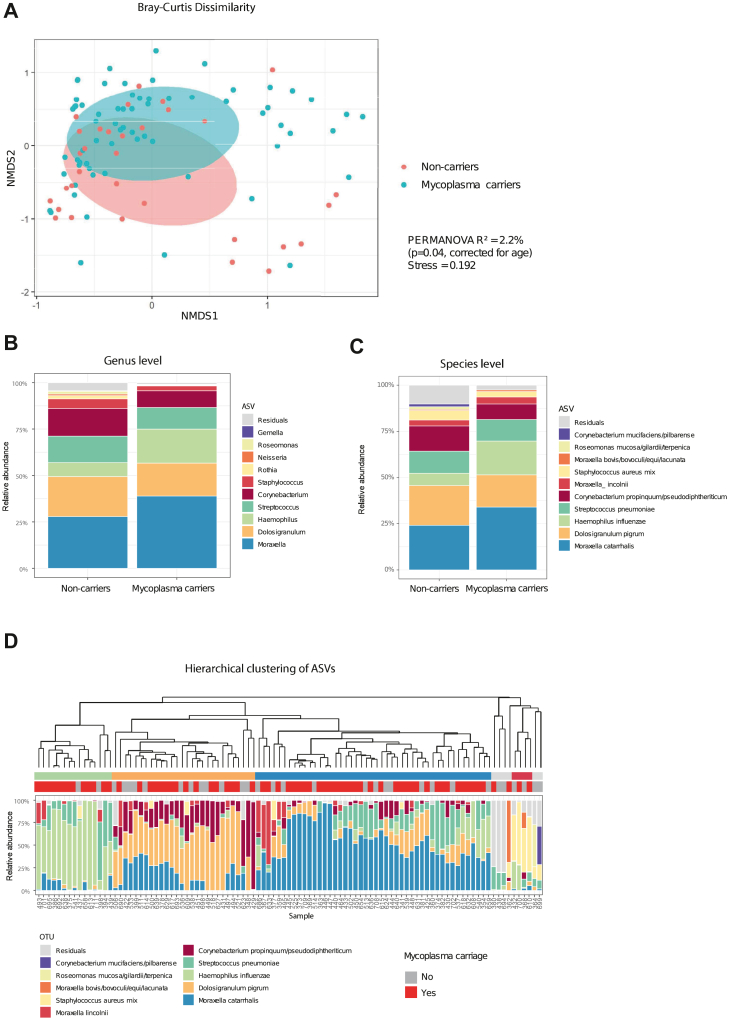
**Microbiota composition of *Mycoplasma pneumoniae* carriers compared to non-carriers with rRTI**s. (A) nMDS plot comparing beta diversity based on Bray–Curtis dissimilarity between *M.pneumoniae* carriers and non-carriers (Stress 0.192). (B) Stacked barplot of highest ranking ASVs (based on mean relative abundance) in *M.pneumoniae* carriers and non-carriers on genus level. (C) Stacked barplot of the highest ranking (based on mean relative abundance) on 99%-similarity species level in *M.pneumoniae* carriers and non-carriers. (D) Hierarchical clustering of nasopharyngeal microbiota on 99%-similarity species.

*M.pneumoniae* carriers had higher bacterial density compared to non-carriers (age-adjusted OR 1.03 [95% CI 1.01–1.07], p = 0.04, Supplementary Figure S3A). Among carriers, there was a positive trend between bacterial density and *M.pneumoniae* copy number (rho 0.23, p = 0.06, Supplementary Figure S3B). Next, we examined within-sample (alpha) diversity using the Shannon index. Children with rRTIs who carried *M.pneumoniae* had lower alpha diversity (age-adjusted OR 0.25 [95% CI 0.09–0.59], p < 0.01, Fig. 3A) and lower richness (number of observed species, age-adjusted OR 0.98 [95% CI 0.96–0.99], p = 0.01, Fig. 3B). While there was a trend towards a positive correlation between alpha diversity and age in non-carriers (rho 0.32, p = 0.07), this correlation was not present in *M.pneumoniae* carriers (rho −0.06, p = 0.61, Fig. 3C). Beta diversity (between-sample diversity) indicated that the NP microbiota composition differed between *M.pneumoniae* carriers and non-carriers (PERMANOVA R^2^ 2.2%, p = 0.04, Fig. 4A).

The most abundant genera in *M.pneumoniae* carriers were *Moraxella*, *Haemophilus*, *Dolosigranulum*, *Streptococcus,* and *Corynebacterium* (Fig. 4B and C). Hierarchical clustering revealed four clusters: *Moraxella catarrhalis*, *Dolosigranulum pigrum* together with *Corynebacterium propinquum/pseudodiphtheriticum*, *Haemophilus influenzae/haemolyticus,* and a *Staphylococcus aureus* cluster (Fig. 4D). The *H. influenzae/haemolyticus* cluster had a higher prevalence of *M.pneumoniae* carriers (87%) compared to the other clusters (72% in *M. catarrhalis* cluster, 56% in *D. pigrum/C. propinquum/pseudodiphtheriticum* cluster, and 50% in *S. aureus* cluster). *M.pneumoniae* carriage was indeed associated with increased *H. influenzae/haemolyticus* RA (age-adjusted OR 10.97 [95% CI 1.29–210.32], p = 0.03) and decreased *C. propinquum/pseudodiphtheriticum* RA (age-adjusted OR 0.03 [95% CI 0.00–1.03], p = 0.05, Supplementary Figure S3C). Since *H. influenzae/haemolyticus* stood out, we subtyped these bacteria with a qPCR in 81 samples. *H. influenzae* was detected in all samples and in 19 samples *Haemophilus haemolyticus* was co-detected with a higher Cq value than *H. influenzae* (median Cq 33.9 [IQR 33.6–34.5] and 22.9 [IQR 21.1–23.7], respectively, p < 0.001). *H. influenzae* concentration determined by qPCR correlated strongly with absolute abundance of *H. influenzae/haemolyticus* determined by 16S-rRNA sequencing (rho 0.87, p < 0.001, Supplementary Figure S3D), whereas this was not the case for *H. haemolyticus*. Thus, we conclude that *H. influenzae* is predominantly present in our dataset.

Finally, we investigated the relationship between antibody levels, major microbiota factors, and *M.pneumoniae* copy number. Neither serum nor mucosal antibody levels, nor alpha diversity, nor absolute abundance of *H. influenzae* were associated with *M.pneumoniae* copy number (Supplementary Figure S4).

### Factors associated with *M.pneumoniae* carriage

To identify the clinical, microbiological, and immunological factors associated with *M.pneumoniae* carriage, we conducted univariable and multivariable logistic regression analysis including age, RTI symptoms during sampling and prophylactic antibiotic use as possible confounding variables. Since serum IgA and total IgG were correlated, we could only include one variable in the multivariable model to prevent multicollinearity. As IgA was measured both systemically and in the respiratory tract, we included serum IgA to compare compartments. In multivariable analysis, *H. influenzae* was strongly associated with *M.pneumoniae* carriage (OR 45.01 [95% CI 2.74–1608.11], p = 0.02) and alpha diversity was negatively associated with *M.pneumoniae* carriage (OR 0.27 [95% CI 0.09–0.67], p < 0.01, Table 2).

**Table 2:** Factors associated with *M.pneumoniae* carriage in children with recurrent respiratory tract infections.

	Univariable analysis		Multivariable analysis	
	Odds ratio [95% CI^c^]	p-value	Odds ratio [95% CI^c^]	p-value
Age in years	0.92 [0.74–1.13]	0.42	0.99 [0.76–1.29]	0.42
RTI^a^ symptoms present at sample collection	1.37 [0.61–3.14]	0.45	0.72 [0.27–1.29]	0.94
Antibiotic prophylaxis 3 months prior to sampling	0.74 [0.25–2.33]	0.58	2.58 [0.63–12.24]	0.20
Normalized mucosal *M.pneumoniae*-specific sIgA levels	1.00 [1.00–1.00]	0.62		
Normalized total mucosal sIgA levels	0.84 [0.46–1.57]	0.57		
Alpha diversity (Shannon index)	**0.22 [0.09**–**0.49]**	**<0.01**	**0.27 [0.09**–**0.67]**	**<0.01**
*Moraxella catarrhalis*^b^	4.42 [0.97–22.91]	0.06	6.37 [0.75–61.20]	0.10
*Haemophilus influenzae*^b^	**24.19 [2.63**–**511.66]**	**0.02**	**45.01 [2.74**–**1608.11]**	**0.02**
*Coryngebacterium propinquum/pseudodiphtheriticum*^b^	**0.03 [0.00**–**0.62]**	**0.03**		
*Streptococcus pneumoniae*^b^	0.45 [0.03–6.69]	0.55		
Serum IgA	3.15 [1.01–11.96]	0.07	4.11 [1.06–19.48]	0.05

The bold part emphasize the statistical significance.

a RTI = Respiratory tract infection.

b Relative abundance.

c CI = Confidence interval

## Discussion

In our study of children with rRTIs and their family members, we found a high prevalence of *M.pneumoniae* carriage of 53%. This was linked to rRTIs, evident by the higher carriage prevalence in affected children compared to their family members without rRTIs. Furthermore, household transmission seemed to elevate the overall *M.pneumoniae* prevalence in this cohort. Our investigation into respiratory viruses and nasopharyngeal microbiota revealed that among children with rRTIs, *M.pneumoniae* carriage was associated with higher co-detection of viruses, lower alpha diversity, and increased *H. influenzae* relative abundance.

In previous studies, the prevalence of *M.pneumoniae* carriage in children ranged from 21% to 56%.^8^^,^^35^ The 68% prevalence of *M.pneumoniae* carriage in children with rRTIs was striking, considering that prevalence rates during *M.pneumoniae* epidemics typically remain below 50%.^8^ Notably, surveillance data indicated no *M.pneumoniae* epidemic during our study period and clinical and/or laboratory signs that could indicate *M.pneumoniae* infection were similar in rRTI children with and without *M.pneumoniae* carriage. Our results thus indicate an increased risk of *M.pneumoniae* carriage in children with rRTIs.

Additionally, we found that *M.pneumoniae* carriage was associated with the total number of family members carrying this bacterium. While previous research in pediatric patients with *M.pneumoniae* infection highlighted household transmission of *M.pneumoniae* carriage, varying rates (15–73%) were observed.^11^^,^^12^ In accordance with these studies, family members included in our study had no RTI symptoms during sampling, indicating asymptomatic carriage. While we could not directly determine the direction of transmission in this cross-sectional cohort, our findings suggest that family members' *M.pneumoniae* carriage related to that of the rRTIs-affected child, rather than the reverse. The cohabitation of family members with rRTIs-affected children could thus explain the elevated prevalence of *M.pneumoniae* carriage in family members without (recurrent) infections.

Investigation of clinical data on RTI symptoms, fever, and inflammatory laboratory markers at time of sampling suggested a low prevalence of *M.pneumoniae* infection in this cohort. Moreover, none of these factors differed between *M.pneumoniae* carriers and non-carriers. As such, we do not believe that potential *M.pneumoniae* infection influenced our findings. Previous studies have suggested associations between allergic predisposition, elevated serum IgE levels, and *M.pneumoniae* infection or its extra-pulmonary manifestations.^36^, ^37^, ^38^ In our cohort of young children with high *M.pneumoniae* carriage, we did not identify any extra-respiratory manifestations of *M. pneumoniae*. Furthermore, allergic predisposition and total serum IgE levels were not associated with *M.pneumoniae* carriage.

We found a higher prevalence of co-detected viruses in *M.pneumoniae* carriers than in non-carriers. This is also observed in studies reporting co-detection of other potential pathogens, predominantly viruses, in 28% of *M.pneumoniae* infections in young children.^39^ Metagenomic analysis of nasopharyngeal aspirates showed that in *M.pneumoniae* CAP patients, the number of RSV reads was higher and influenza virus was detected more frequently compared to non-*M.pneumoniae* CAP patients,^40^ whereas in our study only human rhinovirus was found more frequently in *M.pneumoniae* carriers. We hypothesize that distinct viral infections could influence both *M. pneumoniae* carriage and the onset of related respiratory diseases.

Antibody responses play a crucial role in immune protection against *M.pneumoniae*.^13^^,^^41^ Our previous research has described the role of *M.pneumoniae-*specific mucosal IgA in preventing bacterial adhesion to respiratory epithelial cells.^13^ However, studies investigating the relationship between antibody deficiency and *M.pneumoniae* have primarily focused on *M.pneumoniae* infection rather than carriage.^41^ Our study describes the prevalence of *M.pneumoniae* carriage in children with rRTIs and the relationship with serum and mucosal antibody levels. Interestingly, we found a negative association between serum antibody deficiency and *M.pneumoniae* carriage. From a mechanistic point of view, it seems unlikely that (partial) antibody deficiencies protect against *M.pneumoniae* carriage and this association is therefore more likely explained by a microbiota dysbiosis driven by low antibody levels, as previously described for lowered IgA levels.^42^ Our observation that children with rRTIs carrying *M.pneumoniae* had similar *M.pneumoniae-*specific antibody levels when compared to non-carriers, together with our findings that carriers and non-carriers did not differ in their clinical presentation and/or laboratory inflammatory markers, emphasized that the prevalence of ongoing *M.pneumoniae* infections was at most minimal in this cohort.

The lack of increased *M.pneumoniae*-specific antibodies in carriers is in line with our previous study where we found that *M.pneumoniae* infection, but not carriage, increased *M.pneumoniae*-specific mucosal antibody levels.^13^ This observation suggests that children with rRTIs are not at increased risk to develop antibody-mediated extra-pulmonary manifestations,^43^^,^^44^ although prevalence of *M.pneumoniae* URT carriage was high.

To our knowledge, the respiratory microbiota composition has not been previously described in relation to *M.pneumoniae* carriage. In our cohort, high abundance of *H. influenzae* and low alpha diversity were associated with *M.pneumoniae* carriage in multivariable analysis. Both factors have been previously described in relation to RTI susceptibility in early life,^45^^,^^46^ as well as acute RTIs.^46^^,^^47^ Other studies investigating the role of the microbiota in relation to *M.pneumoniae* have focused on patients with *M.pneumoniae* pneumonia.^48^, ^49^, ^50^ Similar to our finding for *M.pneumoniae* carriage, these studies described a lower alpha diversity in the microbiota of children with *M.pneumoniae* infection compared to children without infection,^49^^,^^50^ as well as a different microbiota composition.^49^ Two studies reported an increased abundance of *Streptococcus* and a lower abundance of *Staphylococcus*, *Dolosigranulum,* and *Corynebacterium* in the NP microbiota of patients with *M.pneumoniae* pneumonia compared to healthy controls,^48^^,^^49^ but did not report a difference for *Haemophilus* abundance. However, one recent study did find that *Haemophilus* species were more abundant in children with severe *M.pneumoniae* pneumonia compared to children with mild infection.^51^

Previous research has identified potential causes of rRTIs such as immunodeficiencies, asthma, and allergies,^52^, ^53^, ^54^ but also microbiota-related factors like lower alpha diversity,^55^ higher levels of Haemophilus spp. and lower levels of Corynebacterium spp. and *D. pigrum*.^19^^,^^55^, ^56^, ^57^ Given that the respiratory microbiota profiles of *M.pneumoniae* carriers also showed lower abundance of RTI health-associated pathogens such as Corynebacterium spp.,^19^^,^^55^, ^56^, ^57^ it is likely that microbial dysbiosis involving multiple bacteria, rather than mere *M.pneumoniae* carriage, may contribute to rRTIs. Future microbiota studies should explicitly consider *M.pneumoniae* as a pathogen of interest in patients with rRTIs and adjust detection methods to incorporate *M.pneumoniae*.

A limitation of our study was that we were unable to detect *M.pneumoniae* by 16S-rRNA sequencing, because the sensitivity of the 16S qPCR for the *M.pneumoniae* 16S gene was lower compared with our specific *M.pneumoniae* qPCR. The 16S gene of *M.pneumoniae* could only be detected when it was present at very high concentrations, that exceeded the biologically expected levels. This indicates that the absence of *M.pneumoniae* in our sequencing dataset cannot be attributed to the primers or pipeline used, but rather to inherent properties of the bacterium itself. *M.pneumoniae* differs from other bacteria in that it lacks a cell wall and has an exceptionally small genome (<1000 kilobases).^58^ Moreover, unlike many bacteria that have multiple copies of the 16S gene, *M.pneumoniae* possesses only a single copy of this gene. These unique features pose challenges for laboratory techniques designed to detect multiple bacteria. This emphasizes the need for a targeted approach, such as the more sensitive *M.pneumoniae*-specific qPCR used in our study, to accurately determine the presence of *M.pneumoniae*. This combination of techniques could provide an appropriate alternative for future research targeting specific bacteria that present challenges in 16S-rRNA sequencing.

Without *M.pneumoniae* in the microbiota analysis, we could not directly assess the relationship between *M.pneumoniae* relative abundance and factors associated with carriage. However, we did evaluate *M.pneumoniae* copy number and found no relationship with antibody levels, alpha diversity, or absolute abundance of *H. influenzae*, which suggests that these factors are associated with the presence of *M.pneumoniae*, but do not directly impact its abundance. Lastly, because of the cross-sectional design of our study, we could not determine whether *H. influenzae* plays a role in susceptibility to *M.pneumoniae* or whether it is enriched in response to *M.pneumoniae* colonization. Our findings show that the presence of *M.pneumoniae* and *H. influenzae* are strongly associated in the URT microbiota. We hypothesize several mechanisms for this co-occurrence. *M.pneumoniae* and *H. influenzae* could be associated with a similar lifestyle that influences microbiota composition. Alternatively, *M.pneumoniae* and *H. influenzae* could prefer a similar micro-environment in the URT or they could directly benefit from each other's presence and/or metabolic end products. Clinical and laboratory assessment within our cohort did not indicate the presence of *M.pneumoniae* infection. However, lower alpha diversity and higher abundance of *H. influenzae* have both been previously reported in relation to *M.pneumoniae* infection and infection severity. Since we observed similar associations between *H. influenzae* and *M.pneumoniae* carriage as described for *M.pneumoniae* infection, we pose that *M.pneumoniae-*related microbiota alterations may already exist prior to infection, potentially contributing to the development of the infection.

When confronted with a young child with rRTIs and/or their household members, clinicians should be aware of the increased prevalence rates of *M.pneumoniae* carriage compared to the general population. While *M.pneumoniae* carriage can be asymptomatic, it forms a reservoir for family transmission and can precede *M.pneumoniae* infection. We found that *H. influenzae* was most strongly associated with *M.pneumoniae* carriage, and previous literature also shows that *H. influenzae* is associated with worse *M.pneumoniae* pneumonia outcome.^51^ We would therefore recommend antibiotic treatment with appropriate coverage of *M.pneumoniae* and *H. influenzae* in case of suspected pneumonia in children with rRTIs and/or their family members. This is important because most guidelines for empiric treatment of CAP recommend oral amoxicillin as the first-choice, which is suboptimal in this case, given the increasing resistance of *H. influenzae* and the inherent resistance of *M.pneumoniae* to β-lactam antibiotics.

In conclusion, our study demonstrates the high prevalence of *M.pneumoniae* carriage in young children with rRTIs and their family members through household transmission. Analysis of the URT microbiota in children with rRTIs revealed lower alpha diversity and overrepresentation of the disease-associated bacterium *H. influenzae* in *M.pneumoniae* carriers. Our findings reveal previously unknown associations between *M.pneumoniae* carriage and local respiratory microbiota members, and shed light on potential host defense mechanisms against *M.pneumoniae.* To prevent associated morbidity in high-risk children with rRTIs and their family members, antibiotic treatment with appropriate coverage of *M.pneumoniae* and *H. influenzae* should be considered in case of suspected pneumonia.

## Contributors

M.K., R.d.G., A.v.R., W.U., and L.V. contributed to the concept and design of the here presented study. M.K. and L.V. were responsible for patient recruitment, sample and data collection, storage, and analysis of clinical data. R.d.G., A.d.B., and S.E. conducted the laboratory analysis and contributed toward data acquisition for *M.pneumoniae* qPCR and ELISA. R.H., K.A., and M.L.C. conducted laboratory work for the microbiota analysis and downstream handling of data was conducted by M.K. and W.d.SP., M.K., R.d.G., W.d.SP., E.v.d.V., J.L., M.B., D.B., A.v.R., W.U., and L.V. contributed toward data interpretation and to writing the manuscript. All authors confirm that they had full access to all the data in the study and accept responsibility to submit for publication.

## Data sharing statement

The microbiota sequencing datasets supporting this article are available at the “NCBI BioSample database PRJNA897835”. All other datasets and codes are available in git repository http://gitlab.com/mkoenen2/mycoplasma_pneumoniae_carriage.

## Declaration of interests

None of the authors have a conflict of interest.

## Appendix A Supplementary data

Supplementary Tables and Figures

Study Protocol
