# Supplementary material for: Mycoplasma pneumoniae carriage in children with recurrent respiratory tract infections is associated with a less diverse and altered microbiota
Source: eBioMedicine. 2023 Nov 10;98:104868. doi: 10.1016/j.ebiom.2023.104868 (PMC10679896; doi:10.1016/j.ebiom.2023.104868)
Supplement: Supplementary Tables and Figures [file mmc1.pdf]

Supplementary Figure 1

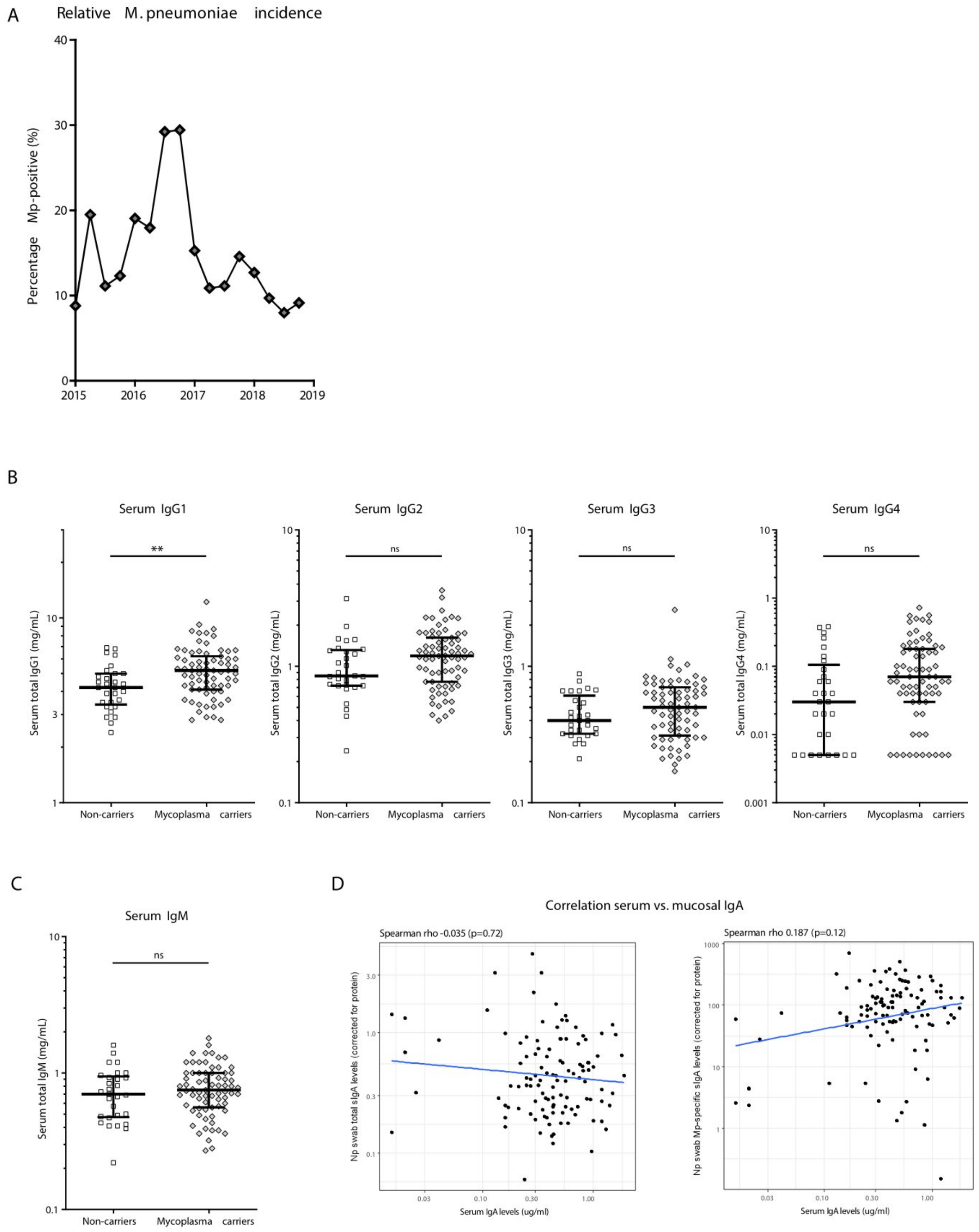

(A) Percentage qPCR positive for *M. pneumoniae* in subjects in the national respiratory tract infection surveillance system (Dutch Working Group on Clinical Virology from the Dutch Society for Clinical Microbiology) in the Netherlands from 2015-2019. (B) Serum IgG subclass levels in children with rRTIs in both *M. pneumoniae* carriers and non-carriers. (C) Serum IgM levels in children with rRTIs in both *M. pneumoniae* carriers and non-carriers. (D) Spearman correlations of serum IgA levels and nasopharyngeal total and *M. pneumoniaeI*-specific IgA levels in children with rRTIs. (B-C) Lines represent medians and error bars show interquartile ranges. *p<0.05, **p<0.01 (logistic regression analysis corrected for age). rRTIs=recurrent respiratory tract infections.

(B) **Supplementary Figure 2**

A

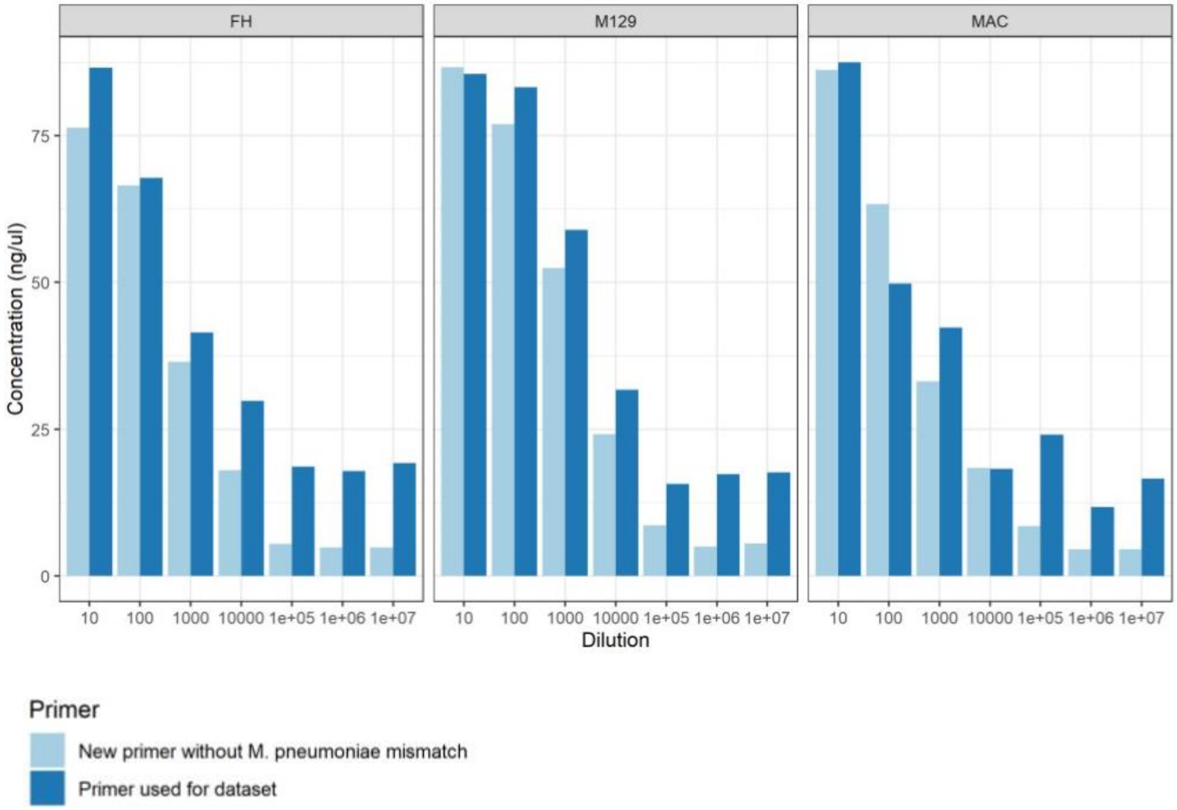

B

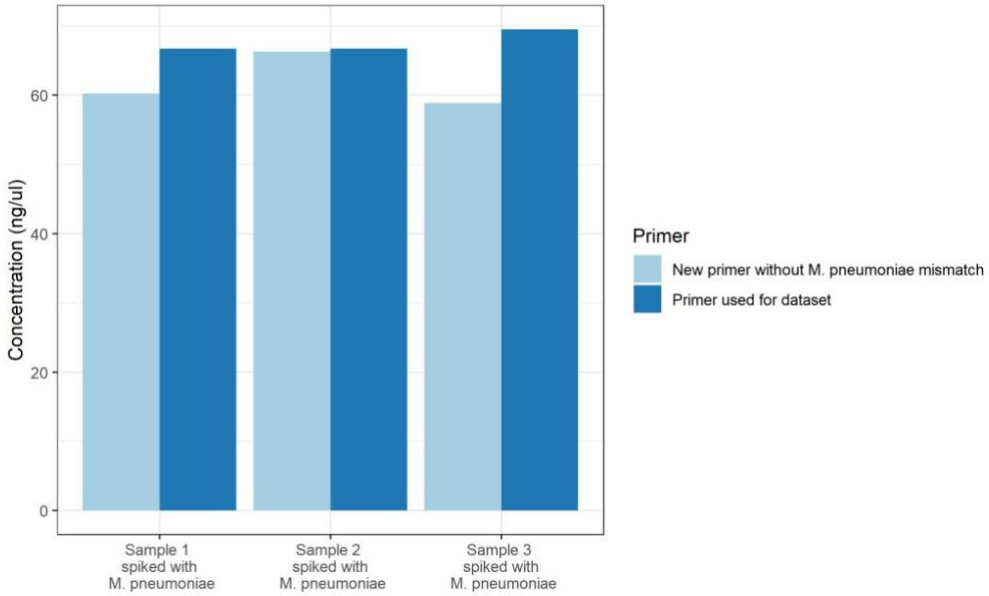

(A) Bacterial density as measured with RT-qPCR of three isolated *M. pneumoniae* strains measured with two different primer sets (B) Three nasopharyngeal swabs postive with *M. pneumoniae* and further spiked with *M. pneumoniae* strains measured with two different primer sets.

Supplementary Figure 3

A

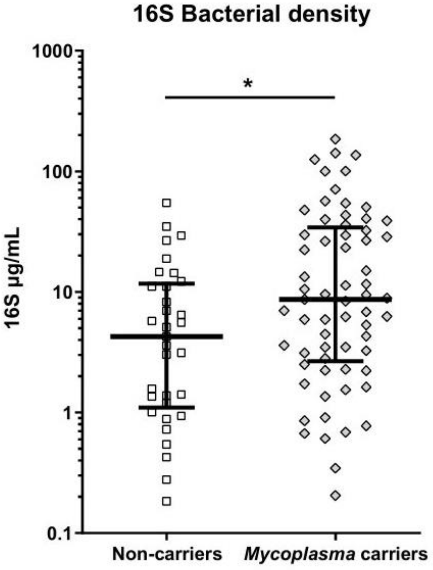

B

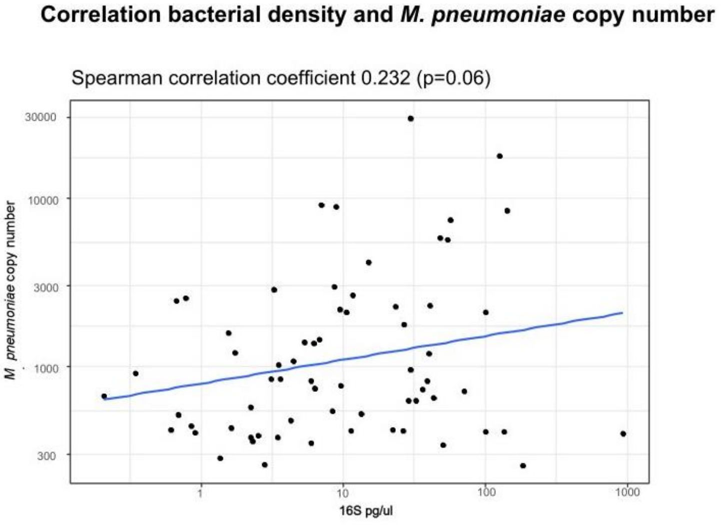

C

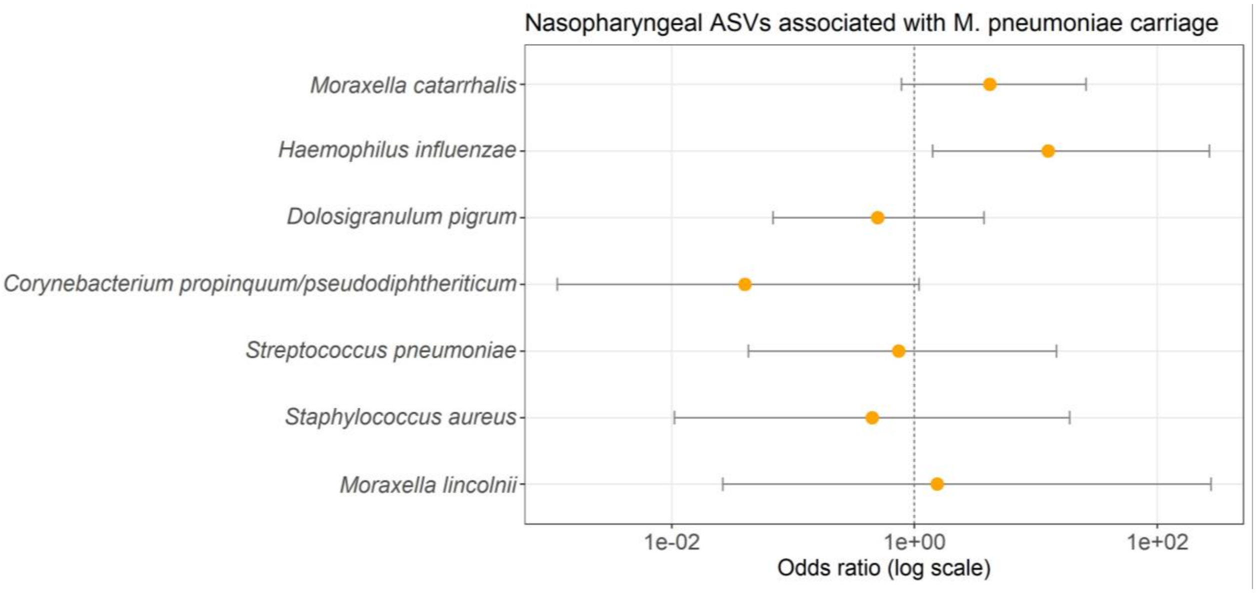

D

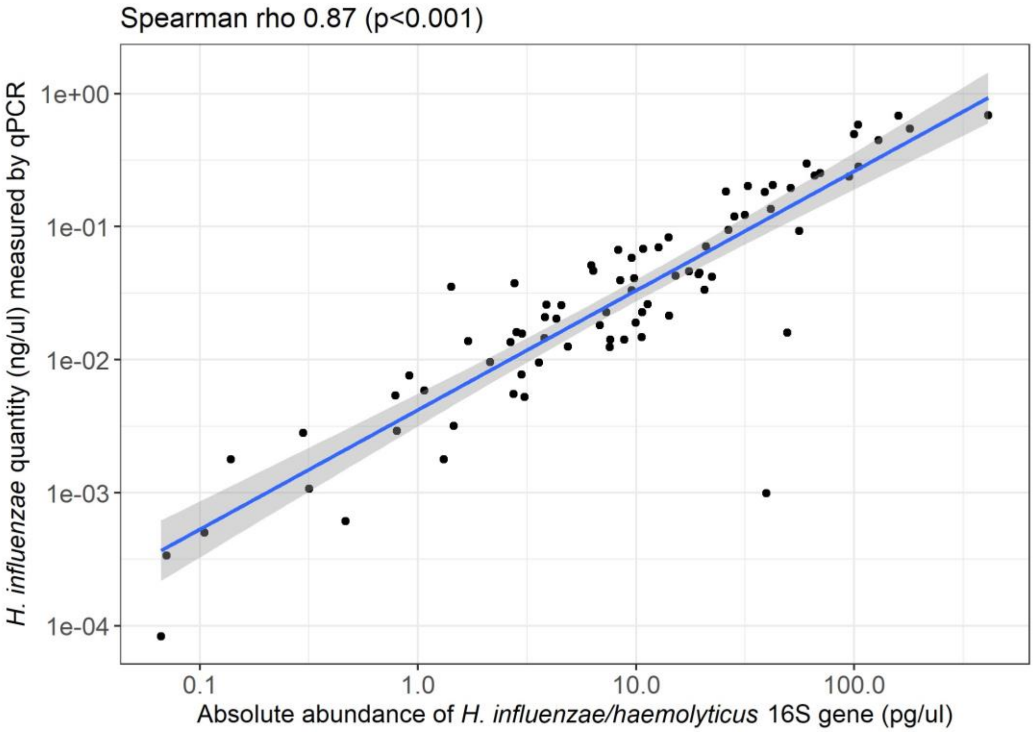

(A) Nasopharyngeal 16S bacterial density in *M. pneumoniae* carriers and non-carriers. (B) Spearman correlations of nasopharyngeal bacterial density with *M. pneumoniae* copy number.

(C) Association (Odds Ratios corrected for age) of highest ranking (based on mean relative abundance) on 99%-similarity species with *M. pneumoniae* carriage. (D) Correlation of *H. influenzae* concentration measured with qPCR and absolute abundance of *H. influenzae/haemolyticus* measured with 16S-rRNA sequencing. Grey shade around line represent 95% confidence intervals.

Supplementary Figure 4

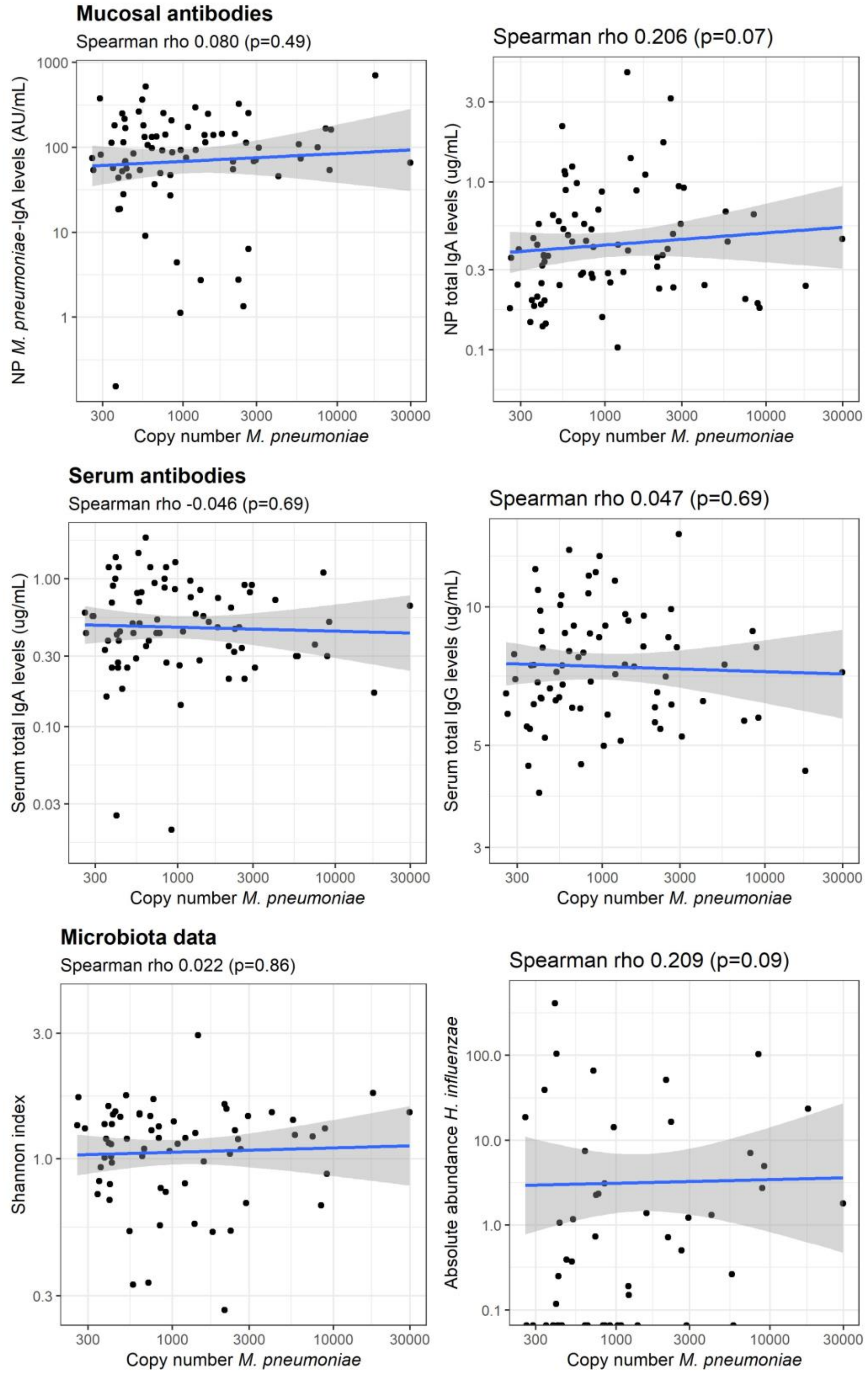

Spearman correlation plots of *M. pneumoniae* copy number in relation to nasopharyngeal *M. pneumoniae*-IgA an total IgA levels (top two plots), serum IgA and IgG levels (middle two plots) and alpha diversity and absolute abundance of *Haemophilus influenzae* (bottom two plots). Grey shade around line represent 95% confidence intervals.

Supplementary Table 1: Primers and probes used for viral panel qPCR

Virus	Primer/ probes	Mod5'	Sequence (5'-3')	Mod3'	Bases	Purification	Molecular weight	Extinction coefficient	% GC	Qty (OD)	Qty (nmol)	to 100 µM	Batch Number Eurogent
Influenza A	forward primer		AAA-GCG-AAT-TTC-AGT-GTG-AT		20	RP-CARTRIDGE	6180	204600	35	17,19	84,03	840,3	7345549
	reverse primer		GAA-GGC-AAT-GTG-AGA-TTT		18	RP-CARTRIDGE	5603	185900	38,9	6,31	33,95	339,5	7345550
	probe	6-FAM	CCC-TCT-TCG-GTG-AAA-GCC-CT	BHQ ®-1	20	HPLC-RP	7121	204600	60	12,28	60,01	600,1	7345573
Influenza B	forward primer		GTC-CAT-CAA-GCT-CCA-GTT-TT		20	RP-CARTRIDGE	6043	183600	45	18,94	103,16	1031,6	7345555
	reverse primer		TCT-TCT-TAC-AGC-TTG-CTT-GC		20	RP-CARTRIDGE	6025	170100	45	18,19	106,91	1069,1	7345556
	probe	Cy 5®	CCT-CCG-TCT-CCA-CCT-ACT-TCG-TT	BHQ ®-2	23	HPLC-RP	7928	209900	56,5	2,89	13,75	137,5	7345576
hMPV	forward primer		AAC-CGT-GTA-CTA-AGT-GAT-GCA-CTC		24	RP-CARTRIDGE	7337	234100	45,8	14,96	63,89	638,9	7345551
	reverse primer		CAT-TGT-TTG-ACC-GGC-CCC-ATA-A		22	RP-CARTRIDGE	6670	205000	50	20,57	100,35	1003,5	7345552
	probe	6-FAM	CTT-TGC-CAT-ACT-CAA-TGA-ACA-AAC	BHQ ®-1	24	HPLC-RP	8357	259600	37,5	10,22	39,37	393,7	7345574

RSV A	forward primer		AGA-TCA-ACT-TCT-GTC-ATC-CAG-CAA		24	RP-CARTRIDGE	7281	233900	41,7	17,99	76,91	769,1	7345553
	reverse primer		TTC-TGC-ACA-TCA-TAA-TTA-GGA-G		22	RP-CARTRIDGE	6734	217900	36,4	15,13	69,44	694,4	7345554
	probe	6-FAM	CAC-CAT-CCA-ACG-GAG-CAC-AGG-AGA-T	BHQ ®-1	25	HPLC-RP	8746	278300	56	10,92	39,23	392,3	7345575
RSV B	forward primer		AAG-ATG-CAA-ATC-ATA-AAT-TCA-CAG-GA		26	RP-CARTRIDGE	8004	276900	30,8	23,28	84,05	840,5	7345557
	reverse primer		TGA-TAT-CCA-GCA-TCT-TTA-AGT-A		22	RP-CARTRIDGE	6709	218600	31,8	11,18	51,12	511,2	7345558
	probe	Cy 5®	TTT-CCC-TTC-CTA-ACC-TGG-ACA-TA	BHQ ®-2	23	HPLC-RP	8000	226500	43,5	3,59	15,85	158,49	7345577
Rhinovirus	forward primer		TGG-ACA-GGG-TGT-GAA-GAG-C		19	RP-CARTRIDGE	5958	194500	57,9	11,72	60,26	602,6	7345559
	reverse primer		CAA-AGT-AGT-CGG-TCC-CAT-CC		20	RP-CARTRIDGE	6062	190600	55	13,6	71,34	713,4	7345560
	probe	HEX	TCC-TCC-GGC-CCC-TGA-ATG	BHQ ®-1	18	HPLC-RP	6710	195400	66,7	12,71	65,06	650,6	7345578
Parainfluenza 1	forward primer		ACC-TAC-AAG-GCA-ACA-ACA-TC		20	RP-CARTRIDGE	6048	200200	45	17,42	86,99	869,9	7345561
	reverse primer		CTT-CCT-GCT-GGT-GTG-TTA-AT		20	RP-CARTRIDGE	6105	178700	45	16,55	92,58	925,8	7345562

	probe	Cy 5®	CAA-ACG-ATG-GCT-GAA-AAA-GGG-A	BHQ ®-2	22	HPLC-RP	7941	250500	45,5	3,25	12,97	129,7	7345579
Parainfluenza 2	forward primer		CCA-TTT-ACC-TAA-GTG-ATG-GAA		21	RP-CARTRIDGE	6429	209800	38,1	14,05	66,95	669,5	7345563
	reverse primer		CGT-GGC-ATA-ATC-TTC-TTT-TT		20	RP-CARTRIDGE	6064	179400	35	16,88	94,07	940,7	7345564
	probe	HEX	AAT-CGC-AAA-AGC-TGT-TCA-GTC-AC	BHQ ®-1	23	HPLC-RP	8315	265300	43,5	14,68	55,32	553,2	7345580
Parainfluenza 3	forward primer		CCA-GGG-ATA-TAY-TAY-AAA-GGC-AAA-A		25	RP-CARTRIDGE	7716	269200	32	28,51	105,92	1059,2	7345565
	reverse primer		CCG-GGR-CAC-CCA-GTT-GTG		18	RP-CARTRIDGE	5509	166700	66,7	24,66	147,91	1479,1	7345566
	probe	6-FAM	TGG-RTG-TTC-AAG-ACC-TCC-ATA-YCC-GAG-AAA	BHQ ®-1	30	HPLC-RP	10292	324700	43,3	12,96	39,93	399,3	7345581
Coronavirus 229E	forward primer		CAG-TCA-AAT-GGG-CTG-ATG-CA		20	RP-CARTRIDGE	6166	198000	50	12,94	65,33	653,3	7345567
	reverse primer		AAA-GGG-CTA-TAA-AGA-GAA-TAA-GGT-ATT-CT		29	RP-CARTRIDGE	9022	310300	31	25,29	81,49	814,9	7345568
	probe	6-FAM	CCC-TGA-CGA-CCA-CGT-TGT-GGT-TCA	BHQ ®-1	24	HPLC-RP	8397	249600	58,3	12,12	48,56	485,6	7345582

Coronavirus NL63	forward primer		ACG-TAC-TTC-TAT-TAT-GAA-GCA-TGA-TAT-TAA		30	RP-CARTRIDGE	9203	305500	26,7	22,8	74,63	746,3	7345571
	reverse primer		AGC-AGA-TCT-AAT-GTT-ATA-CTT-AAA-ACT-ACG		30	RP-CARTRIDGE	9197	305800	30	25,24	82,55	825,5	7345572
	probe	HEX	ATT-GCC-AAG-GCT-CCT-AAA-CGT-ACA-GGT-GTT	BHQ ®-1	30	HPLC-RP	10505	329000	46,7	15,14	46,01	460,1	7345584
Coronavirus OC43	forward primer		CGA-TGA-GGC-TAT-TCC-GAC-TAG-GT		23	RP-CARTRIDGE	7080	222400	52,2	21,25	95,54	955,4	7345569
	reverse primer		CCT-TCC-TGA-GCC-TTC-AAT-ATA-GTA-ACC		27	RP-CARTRIDGE	8154	251100	44,4	23,94	95,35	953,5	7345570
	probe	Cy 5®	TCC-GCC-TGG-CAC-GGT-ACT-CCC-T	BHQ ®-2	22	HPLC-RP	7713	206400	68,2	3,46	16,74	167,4	7345583

Supplementary Table 2: Study demographics of family members stratified by *M. pneumoniae* carriage status

A Siblings

	**Siblings that are *M. pneumoniae* carriers (n=14)**	**Siblings that are non-carriers (n=16)**	**p-value**
Age in years (median [IQR*])	6.5 [4.9-10.5]	5.8 [4.3-8.2]	p=0.47
Female	57% (8/14)	50% (8/36)	p=0.98
Recurrent pneumonia	0% (0/14)	0% (0/16)	NA‡
IgA deficiency	14% (2/14)	13% (2/16)	p=0.88
Sampled in winter	0% (0/14)	0% (0/16)	NA‡
RTI† symptoms at time of sampling	0% (0/14)	0% (0/16)	NA‡
Number of times the same NP§ swab was analyzed per subject			p=0.08
1 time analyzed	29% (4/14)	63% (10/16)	
2 times analyzed	71% (10/14)	38% (6/16)	

B Parents

	**Parents that are *M. pneumoniae* carriers (n=17)**	**Parents that are non-carriers (n=45)**	**p-value**
Age in years (median [IQR*])	39.3 [33.5-41.7]	38.4 [31.9-42.1]	p=0.65
Female	59% (10/17)	60% (27/45)	p=1.00
IgA deficiency	12% (2/17)	9% (4/45)	p=0.81
Sampled in winter	6% (1/17)	13% (6/45)	p=0.66
RTI† symptoms at time of sampling	0% (0/17)	0% (0/45)	NA‡
Number of times the same NP§ swab was analyzed per subject			NA‡
1 time analyzed	100% (17/17)	100% (45/45)	

* IQR = interquartile range
† RTI= respiratory tract infection
‡ NA = Not applicable
§ NP = nasopharyngeal

Supplementary Table 3: Study demographics of children with rRTIs differentiated based on whether RTI symptoms were present at time of sampling

	**Acute RTI at time of sampling (n=23)**	**No acute RTI at time of sampling (n=78)**	**p-value**
M. pneumoniae carriage	70% (16/23)	68% (53/78)	p=1.00
Age in years (median [IQR*])	3.5 [2.8-5.2]	4.0 [2.5-5.5]	p=0.21
Female sex assigned at birth	39% (9/23)	45% (35/78)	p=0.80
Recurrent pneumonia	18% (4/22)	18% (14/76)	p=0.98
Any virus detected	31% (4/13) §	30% (8/27) §	p=1.00
Rhinovirus	23% (3/13)	22% (6/27)	
Rhinovirus and parainfluenza virus 1	8% (1/13)	0% (0/27)	
Parainfluenza virus 2	0% (0/13)	4% (1/27)	
Coronavirus NL63	0% (0/13)	4% (1/27)	
Any prophylactic antibiotics prior to sampling	35% (8/23)	29% (22/77)	p=0.76
Co-trimoxazole	26% (6/23)	23% (18/77)	
Amoxicillin	9% (2/23)	3% (2/77)	
Amoxicillin/clavulanic acid	0% (0/23)	1% (1/77)	
Trimethoprim	0% (0/23)	1% (1/77)	
Sampled in winter	17% (4/23)	19% (15/78)	p=1.00
Fever at time of sampling	23% (5/22)	1% (1/76)	P<0.01
Extra-pulmonary manifestations of *M. pneumoniae*	0% (0/23)	0% (0/78)	NA
Asthma/recurrent wheezing	59% (9/22)	49% (33/68)	p=0.54
Allergic rhinitis	62% (13/22)	49% (32/65)	p=0.45
Allergic dermatitis	50% (11/22)	50% (34/68)	p=1.00
Food allergy	36% (8/22)	21% (14/68)	p=0.23
Autoimmune disease	0% (0/23)	1% (1/78)<br>Celiac disease (n=1)	p=1.00
Hemoglobulin (mmol/L, median [IQR*])	7.3 [7.2-8.1]	7.6 [7.4-8.0]	p=0.58¶
Erythrocytes (count x10^{12}/L, median [IQR*])	4.5 [4.3-4.9]	4.65 [4.3-4.8]	p=0.60¶
C-reactive protein (mg/L, median [IQR*])	1.0 [0.5-4.0]	0.5 [0.5-1.0]	p=0.59¶
White blood cell (count x10^9/L, median [IQR*])	10.6 [7.4-11.8]	10.0 [8.5-11.6]	p=0.70 ¶

Lymphocytes (count x10^9/L, median [IQR*])	4.4 [3.4-6.0]	4.4 [3.0-5.3]	p=0.89 [¶]
Monocytes (count x10^9/L, median [IQR*])	0.58 [0.44-0.76]	0.68 [0.57-0.79]	p=0.48 [¶]
Neutrophilic granulocytes (count x10^9/L, median [IQR*])	3.5 [2.5-5.7]	3.8 [3.2-5.5]	p=0.61 [¶]
Basophilic granulocytes (count x10^9/L, median [IQR*])	0.04 [0.00-0.08]	0.06 [0.03-0.09]	p=0.78 [¶]
Eosinophilic granulocytes (count x10^9/L, median [IQR*])	0.23 [0.13-0.39]	0.24 [0.16-0.44]	p=0.51 [¶]
Immunoglobulin E (kU/L, median [IQR*])	21 [7-94]	32 [12-103]	p=0.49 [¶]
Immunoglobulin G (g/L, median [IQR*])	6.7 [5.9-8.0]	7.0 [5.9-8.6]	p=0.98 [¶]
Immunoglobulin A (g/L, median [IQR*])	0.50 [0.41-0.63]	0.51 [0.43-0.63]	p=0.99 [¶]
Immunoglobulin A (g/L, median [IQR*])	0.69 [0.50-0.76]	0.81 [0.55-1.00]	p=0.22 [¶]

Supplementary methods

<u>DIMER-Mycoplasma study (sub-study of the PID study)</u>

The DIMER (Deficiency of IgA and Microbiome in Respiratory tract infections) study, conducted between March 2016 and December 2019, was a prospective cohort study conducted in two hospitals in The Netherlands (Erasmus MC and Wilhelmina Children's Hospital) conducted as part of the PID (Primary Immune Deficiency) study. The DIMER-mycoplasma study was a sub study of the DIMER study with a cross-sectional study design that compared the prevalence of primary antibody deficiencies and *M. pneumoniae* carriage in children with rRTIs to controls without infections. In addition, we aimed to investigate the mucosal immune system, including (*M. pneumoniae-specific*) mucosal antibody levels and the nasopharyngeal microbiota composition, in children with rRTIs with and without *M. pneumoniae* carriage.

<u>Ethical approval</u>

Ethical approval was obtained from the Medical Ethical Committee of the Erasmus MC on 19-06-2013 (METC:NL40331.078). Local approval from the Wilhelmina Children's Hospital, part of the UMC Utrecht was

obtained on 14-02-2014. Legal guardians signed informed consent for participation and the study was carried out in accordance with The Code of Ethics of the World Medical Association (Declaration of Helsinki).

Children with recurrent respiratory tract infections

Children aged six weeks to eight years who were referred to one of the participating hospitals for rRTIs with an indication for immunological screening, were included in the DIMER study. rRTIs were defined according to the guideline of the Dutch Section of Pediatric Infectious Diseases & Immunology and as described by Gruber et al. (1, 2), as: $\geq$11 upper RTIs a year for children up to two years, $\geq$8 upper RTIs a year for children aged between two and five years, $\geq$6 upper RTIs a year for children aged between five and eight years, or $\geq$2 pneumonia episodes diagnosed in one year or $\geq$3 pneumonia episodes diagnosed during the lifetime of the child. The choice for immunological screening was based on the physician's professional opinion after the clinical consultation and physical examination. Furthermore, children with an already known IgA deficiency (2SD below age-appropriate reference values, see Table) diagnosed within the year before inclusion, were also included. Exclusion criteria for the study were: known primary immunodeficiencies requiring immunoglobulin substitution, secondary immunodeficiencies, major congenital anomalies, antibiotic use four weeks prior to inclusion and/or azithromycin prophylaxis within three months prior to sampling (as this could influence *M. pneumoniae* carriage).

Controls (family members of children with rRTIs)

We also included family members of children with rRTIs, living in the same household, as controls in the study. Exclusion criteria for these controls were: known immunodeficiency (except IgA deficiency), rRTIs, secondary immunodeficiencies, major congenital anomalies and/or antibiotic use four weeks prior to inclusion.

Sample collection

We collected serum and nasopharyngeal (NP) swabs from all study participants. All samples were collected by trained study personnel and placed on dry ice immediately after collection. For children with rRTIs, blood was only collected when there was a clinical indication for blood drawing. Hematological and immunological results from laboratory testing conducted at inclusion were extracted from the electronic patient files and C-reactive protein was additionally measured on ROCHE C8000 (module C702) according to manufacturer instruction. NP swabs were only collected when there was no antibiotic use in the prior month. NP swabs were taken in the

nasopharyngeal cavity and rotated clockwise three times and counterclockwise three times, before being stored in RNA protect medium (Qiagen). All samples were transported on dry ice and stored at -80 degrees Celsius until further processing.

Nasopharyngeal swabs were used for respiratory microbiota measurement with 16S-rRNA sequencing and for the detection of *M. pneumoniae* and viruses (viral panel of 12 respiratory viruses) with qPCR. Viral qPCR was only performed in a subset of children of whom a NP swab was collected at the start of winter.

In addition, total IgA and *M. pneumoniae*-specific IgA were measured in the nasopharyngeal swab using ELISA (see below). Serum was used to measure serum IgA, IgG, IgG subclasses, and IgM levels.

<u>Immunoglobulin measurements in serum</u>

Serum IgA, IgM, IgG, and IgG subclass levels were measured as part of routine clinical care. Total IgA, IgG and IgM were measured using nephelometric assay on AU5811 ® (Beckman Coulter, Brea, USA) and IgG subclasses were measured using turbidimetric assay (SPAplus ®, The Biding Site, Thermo Fisher Scientific, Waltham, USA), both with associated kits following manufacturer's guidelines. Total IgE was measured on an Immuncap 250 analyzer according to manufacturer protocol (Thermo Fisher Scientific, Waltham, USA). An antibody deficiency was defined as immunoglobulin (isotype) levels $\leq$-2SD below age-appropriate reference values as used in standard Dutch clinical care:

Reference values for IgA and IgG: range from -2SD to +2SD for different ages.

**Age**	**IgA (g/l)**	**IgG (total) (g/l)**	**IgM (g/l)**
0–2 weeks	<0.16	6.5-12.6	0.03-0.24
0.5–4 months	0.06-0.57	2.6-7.8	0.10-0.55
4–6 months	0.08-0.90	2.2-11.3	0.07-0.65
6–12 months	0.16-0.98	2.6-15.2	0.17-1.20
1–2 years	0.19-1.10	2.6-13.9	0.10-0.87
2–3 years	0.19-2.30	4.3-13.0	0.21-0.87
3–6 years	0.55-2.20	5.2-13.4	0.24-1.80
6–9 years	0.54-2.50	5.2-14.3	0.28-1.90
9–12 years	0.62-3.00	0.1-1.6	5.20-15.60
12–16 years	0.70-3.60	0.3-2.4	5.20-15.60
Adults	0.70-4.00	0.4-2.3	7.00-16.00

Reference values for IgG subclasses: range from -2SD to +2SD for different ages.

Age	IgG1 (g/l)	IgG2 (g/l)	IgG3 (g/l)	IgG4 (g/l)
0-1 months	2.4-10.6	0.87-4.10	0.14-0.55	0.04-0.56
1-4 months	1.8-6.7	0.38-2.10	0.14-0.70	0.02-0.36
4-6 months	1.8-7.0	0.34-2.10	0.15-0.80	0.02-0.23
0.5-1 years	2.0-7.7	0.34-2.30	0.15-0.97	0.01-0.43
1-1.5 years	2.5-8.2	0.38-2.40	0.15-1.07	0.01-0.62
1.5-2 years	2.9-8.5	0.45-2.60	0.15-1.13	0.01-0.79
2-3 years	3.2-9.0	0.52-2.80	0.14-1.20	0.01-1.06
3-4 years	3.5-9.4	0.63-3.00	0.13-1.26	0.02-1.27
4-6 years	3.7-10.0	0.72-3.40	0.13-1.33	0.02-1.58
6-9 years	4.0-10.8	0.85-4.10	0.13-1.42	0.02-1.89
9–12 years	4.0-11.5	0.98-4.80	0.15-1.49	0.03-2.10
12–16 years	3.7-12.8	1.06-6.10	0.18-1.63	0.03-2.30
Adults	4.9-11.4	1.50-6.40	0.20-1.10	0.08-1.40

Immunoglobulin measurements in nasopharyngeal swabs

Total and *M. pneumoniae*-specific IgA levels were measured using an in-house ELISA assay. 96-wells plates (Maxisorp, Corning Costar, Corning, New York, USA, Product no 3369) were coated overnight with *M. pneumoniae* M129 (ATCC 29342) cell lysate for the detection of *M. pneumoniae*-specific antibodies or with anti-human universal immunoglobulin (SouthernBiotech, Birmingham, AL, USA, RRID:AB_2795681) for the detection of total IgA levels. After blocking (PBS-BSA), NP swabs diluted in 0,1% BSA/PBS were incubated overnight. Standards for *M. pneumoniae*-specific IgA were created by making a twofold dilution series of *M. pneumoniae*-IgA positive controls (Virion\Serion GmbH, Wurzburg, Germany, Product no BC127A). Undiluted positive controls were set to 100 Arbitrary Units. Purified IgA (InvivoGen, San Diego, CA, USA) was used to create total IgA standards. Goat anti-human IgA-Alkaline Phosphatase (Sigma, RRID:AB_437951) was used as secondary antibody. 3,3′,5,5′-Tetramethylbenzidine (TMB, Sigma-Aldrich, Product no CL07) was used as a substrate. Optical density was measured at 450 nm and 405 nm using SpectraMax iD3 (Molecular Devices, San José, USA). Antibody levels were normalized to NP swab total protein to account for sampling variation. Total protein levels were measured using CBQCA Protein Quantitation Kit (Thermo Fisher Scientific, Waltham, MA, USA, Product no C6667).

Detection of *M. pneumoniae*, *H. influenzae* and viruses via quantitative polymerase chain reaction (qPCR)

DNA was isolated from NP swabs using QIAamp DNA mini kit (QIAGEN, Product no 51304) and *M. pneumoniae* DNA was detected with in-house real-time qPCR (RT-qPCR) and *M. pneumoniae* DNA load was

quantified using a plasmid dilution series as previously described (3). In brief, a Taqman assay was used to amplify the conserved region of the MPN141 gene. As a standard, a dilution series of pBluescript SK-vector containing the *M. pneumoniae* target sequence was used. In total, three batches of samples were analyzed. In each batch, samples from the previous batch(es) were also reanalyzed, resulting in some samples being analyzed multiple times. Bacterial loads are expressed as *M. pneumoniae* genome copies per mL original sample. If a child had a positive result (>100 copies/mL) in their collected NP swab in any of the times analyzed, they were considered a *M. pneumoniae* carrier.

A subset of the nasopharyngeal samples was also analyzed by real-time qPCR using species- and type-specific primers/probes was used to detect 12 respiratory viruses (Table S1) according to manufacturer's instructions.

All NP swab with a relative abundance of ≥10% as found with 16S-rRNA sequencing were used to subtype *Haemophilus* with hypD and siaT primers (hypD-F GGCAATCAGATGGTTTACAACG, hypD-R CAGCTTAAAGYAAGYAGTGAATG, siaT-F AATGCGTGATGCTGGTTATGAC, siaT-R AAGAGTTTTGCGATAGATTCATTGG), as described previously (4). Genomic DNA from *H. influenzae* strain 86-028NP (GenBank accession no. CP000057.2) and *H. haemolyticus* strain 33390 (GenBank accession no. JTLY01000001) served as positive controls and H2O as a negative control. qPCR was conducted using SSO SYBR Green (Bio-Rad) with a final volume of 1 mL, containing 0.4 mM of each primer and 1 mL template DNA. Thermocycling consisted of 96˚C for 5 min, followed by 40 cycles 96˚C for 5 sec and 60˚C for 20 sec, followed by a melting curve using Bio-Rad CFX96. Sample with a Cq value below 35 were considered positive.

Clinical data

At inclusion, (caretakers of) subjects were asked to fill in a questionnaire on their medical history, underlying conditions, and medication use. Furthermore, a longer questionnaire on all medical history, allergies, and asthma was sent to all caretakers of children with rRTIs. We defined asthma, allergic rhinitis, and allergic dermatitis as described in the International Study of Asthma and Allergies in Childhood questionnaires (5, 6). In addition, length and weight were measured and a physical examination took place at inclusion. Lab results from haematological and immunological screening were extracted from the electronic medical file. RTI symptoms at the time of sampling were registered as categorized as follows:

1) No symptoms
2) Mild RTI (only 1 RTI symptom (see below) or >1 RTI symptom for 1 day only)
3) Moderate RTI without antibiotic use: ≥2 RTI symptoms for ≥2 days without antibiotic use
4) Moderate RTI with antibiotic use: ≥2 RTI symptoms for ≥2 days with antibiotic use
5) Severe RTI: hospital admission because of RTI

We defined an acute RTI episode as a score of three or higher.

RTI symptoms registered:

a) fever (> 38.0 degrees)
b) rhinitis
c) otitis
d) pharyngitis/sore throat
e) hoarseness
f) coughing
g) wheezing
h) dyspnea
i) agitation/crying

References Supplementary Materials

1. Gruber C, Keil T, Kulig M, Roll S, Wahn U, Wahn V, et al. History of respiratory infections in the first 12 yr among children from a birth cohort. Pediatr Allergy Immunol. 2008;19(6):505-12.
2. Driessen GJA. Evidence-based richtlijn diagnostiek naar onderliggende aandoeningen bij kinderen met recidiverende luchtweginfecties: Erasmus MC, Sophia Kinderziekenhuis, subafdeling kinderinfectieziekten-immunologie; 2016 [Available from: https://www.kinderinfectieziekten.nl/wp-content/uploads/2016/09/12-07-2016-Diagnostiek-recidiverende-luchtweginfecties.pdf.
3. Spuesens EB, Hoogenboezem T, Sluijter M, Hartwig NG, van Rossum AM, Vink C. Macrolide resistance determination and molecular typing of Mycoplasma pneumoniae by pyrosequencing. J Microbiol Methods. 2010;82(3):214-22.
4. Price EP, Harris TM, Spargo J, Nosworthy E, Beissbarth J, Chang AB, et al. Simultaneous identification of Haemophilus influenzae and Haemophilus haemolyticus using real-time PCR. Future Microbiol. 2017;12:585-93.
5. Asher MI, Keil U, Anderson HR, Beasley R, Crane J, Martinez F, et al. International Study of Asthma and Allergies in Childhood (ISAAC): rationale and methods. Eur Respir J. 1995;8(3):483-91.
6. Kansen HM, Lebbink MA, Mul J, van Erp FC, van Engelen M, de Vries E, et al. Risk factors for atopic diseases and recurrent respiratory tract infections in children. Pediatr Pulmonol. 2020;55(11):3168-79.

Supplementary information

A. IUPAC Ambiguity Code

IUPAC Code	Meaning	Complement
**A**	A	T
**C**	C	G
**G**	G	C
**T/U**	T	A
**M**	A or C	K
**R**	A or G	Y
**W**	A or T	W
**S**	C or G	S
**Y**	C or T	R
**K**	G or T	M
**V**	A or C or G	B
**H**	A or C or T	D
**D**	A or G or T	H
**B**	C or G or T	V
**N**	G or A or T or C	N

Original sequences: 515F(Caporaso) – 806R (Caporaso), reverse-barcoded:

FWD: GTGCCAGC**M**GCCGCGGTAA

REV: GGACTAC**HV**GGGT**W**TCTAAT

- Reversed complement: ATTAGA**W**ACCC**BD**GTAGTCC

Mismatch

Source: SILVA database v138 Version 2.

Forward: *S. aureus* and *S. pneumonia*: GTGCCAGCAGCCGCGGTAA 100% match

CP010547.118272.119807 M. *pneumoniae* M1139, CP010544.118311.119846 *M. pneumoniae* 85084 and *M. pneumoniae* NRBC 1440185084: GTGCCAGCAGTCGCGGTAA, 1 mismatch

Backward: *S. aureus*, *S. pneumonia*, CP010547.118272.119807 M. *pneumoniae* M1139, CP010544.118311.119846 *M. pneumoniae* 85084 and *M. pneumoniae* NRBC 1440185084: ATTAGATACCCTGGTAGTCC 100% match

B. Full sequences

***Staphylococcus aureus* (control, found in dataset)**

TTTATGGAGAGTTTGATCCTGGCTCAGGATGAACGCTGGCGGCGTGCCTAATACATGCAAGTCGAG
CGAACGGACGAGAAGCTTGCTTCTCTGATGTTAGCGGCGGACGGGTGAGTAACACGTGGATAACCT
ACCTATAAGACTGGGATAACTTCGGGAAACCGGAGCTAATACCGGATAATATTTTGAACCGCATGG

TTCAAAAGTGAAAGACGGTCTTGCTGTCACTTATAGATGGATCCGCGCTGCATTAGCTAGTTGGTA
AGGTAACGGCTTACCAAGGCAACGATGCATAGCCGACCTGAGAGGGTGATCGGCCACACTGGAAC
TGAGACACGGTCCAGACTCCTACGGGAGGCAGCAGTAGGGAATCTTCCGCAATGGGCGAAAGCCT
GACGGAGCAACGCCGCGTGAGTGATGAAGGTCTTCGGATCGTAAAACTCTGTTATTAGGGAAGAA
CATATGTGTAAGTAACTGTGCACATCTTGACGGTACCTAATCAGAAAGCCACGGCTAACTACGTGC
CAGCAGCCGCGGTAATACGTAGGTGGCAAGCGTTATCCGGAATTATTGGGCGTAAAGCGCGCGTA
GGCGGTTTTTTAAGTCTGATGTGAAAGCCCACGGCTCAACCGTGGAGGGTCATTGGAAACTGGAAA
ACTTGAGTGCAGAAGAGGAAAGTGGAATTCCATGTGTAGCGGTGAAATGCGCAGAGATATGGAGG
AACACCAGTGGCGAAGGCGACTTTCTGGTCTGTAACTGACGCTGATGTGCGAAAGCGTGGGGATCA
AACAGGATTAGATACCCTGGTAGTCCACGCCGTAAACGATGAGTGCTAAGTGTTAGGGGGTTTCCG
CCCCTTAGTGCTGCAGCTAACGCATTAAGCACTCCGCCTGGGGAGTACGACCGCAAGGTTGAAACT
CAAAGGAATTGACGGGGACCCGCACAAGCGGTGGAGCATGTGGTTTAATTCGAAGCAACGCGAAG
AACCTTACCAAATCTTGACATCCTTTGACAACTCTAGAGATAGAGCCTTCCCCTTCGGGGGACAAA
GTGACAGGTGGTGCATGGTTGTCGTCAGCTCGTGTCGTGAGATGTTGGGTTAAGTCCCGCAACGAG
CGCAACCCTTAAGCTTAGTTGCCATCATTAAGTTGGGCACTCTAAGTTGACTGCCGGTGACAAACC
GGAGGAAGGTGGGGATGACATCAAATCATCATGCCCCTTATGATTTGGGCTACACACGTGCTACAA
TGGACAATACAAAGGGCAGCGAAACCGCGAGGTCAAGCAAATCCCATAAAGTTGTTCTCAGTTCG
GATTGTAGTCTGCAACTCGACTACATGAAGCTGGAATCGCTAGTAATCGTAGATCAGCATGCTACG
GTGAATACGTTCCCGGGTCTTGTACACACCGCCCGTCACACCACGAGAGTTTGTAACACCCGAAGC
CGGTGGAGTAACCTTTTAGGAGCCAGCCGTCGAAGGTGGGACAAATGATTGGGGTGAAGTCGTAA
CAAGGTAGCCGTATCGGAAGGTGCGGCTGGATCACCTCCTTT

***Streptococcus pneumoniae* (control, found in dataset)**

GACGAACGCTGGCGGCGTGCCTAATACATGCAAGTAGAACGCTGAAGGAGGAGCTTGCTTCTCTG
GATGAGTTGCGAACGGGTGAGTAACGCGTAGGTAACCTGCCTGGTAGCGGGGGATAACTATTGGA
AACGATAGCTAATACCGCATAAGAGTAGATGTTGCATGACATTTGCTTAAAAGGTGCACTTGCATC
ACTACCAGATGGACCTGCGTTGTATTAGCTAGTTGGTGGGGTAACGGCTCACCAAGGCGACGATAC
ATAGCCGACCTGAGAGGGTGATCGGCCACACTGGGACTGAGACACGGCCCAGACTCCTACGGGAG
GCAGCAGTAGGGAATCTTCGGCAATGGACGGAAGTCTGACCGAGCAACGCCGCGTGAGTGAAGAA
GGTTTTCGGATCGTAAAGCTCTGTTGTAAGAGAAGAACGAGTGTGAGAGTGGAAAGTTCACACTGT
GACGGTATCTTACCAGAAAGGGACGGCTAACTACGTGCCAGCAGCCGCGGTAATACGTAGGTCCC

GAGCGTTGTCCGGATTTATTGGGCGTAAAGCGAGCGCAGGCGGTTAGATAAGTCTGAAGTTAAAG

GCTGTGGCTTAACCATAGTAGGCTTTGGAAACTGTTTAACTTGAGTGCAAGAGGGGAGAGTGGAAT

TCCATGTGTAGCGGTGAAATGCGTAGATATATGGAGGAACACCGGTGGCGAAAGCGGCTCTCTGG

CTTGTAACTGACGCTGAGGCTCGAAAGCGTGGGGAGCAAACAGGATTAGATACCCTGGTAGTCCA

CGCTGTAAACGATGAGTGCTAGGTGTTAGACCCTTTCCGGGGTTTAGTGCCGTAGCTAACGCATTA

AGCACTCCGCCTGGGGAGTACGACCGCAAGGTTGAAACTCAAAGGAATTGACGGGGGCCCGCACA

AGCGGTGGAGCATGTGGTTTAATTCGAAGCAACGCGAAGAACCTTACCAGGTCTTGACATCCCTCT

GACGACTCTAGAGATAGAGTTTTCCTTCGGGACAGAGGTGACAGGTGGTGCATGGTTGTCGTCAGC

TCGTGTCGTGAGATGTTGGGTTAAGTCCCGCAACGAGCGCAACCCCTATTGTTAGTTGCCATCATTT

AGTTGGGCACTCTAGCGAGACTGCCGGTAATAAACCGGAGGAAGGTGGGGATGACGTCAAATCAT

CATGCCCCTTATGACCTGGGCTACACACGTGCTACAATGGCTGGTACAACGAGTCGCAAGCCGGTG

ACGGCAAGCTAATCTCTTAAAGCCAGTCTCAGTTCGGATTGTAGGCTGCAACTCGCCTACATGAAG

TCGGAATCGCTAGTAATCGCGGATCAGCACGCCGCGGTGAATACGTTCCCGGGCCTTGTACACACC

GCCCGTCACACCACGAGAGTTTGTAACACCCGAAGTCGGTGAGGTAACCGTAAGGAGCCAGCCGC

CTAAGGTGGGATAGATGATTGGGGTGAAG

C. *Mycoplasma pneumoniae*

>CP010547.118272.119807 Mycoplasma pneumoniae M1139

AATCTGTCAATTTTTCTGAGAGTTTGATCCTGGCTCAGGATTAACGCTGGCGGCATGCCTAATACAT

GCAAGTCGATCGAAAGTAGTAATACTTTAGAGGCGAACGGGTGAGTAACACGTATCCAATCTACCT

TATAATGGGGGATAACTAGTTGAAAGACTAGCTAATACCGCATAAGAACTTTGGTTCGCATGAATC

AAAGTTGAAAGGACCTGCAAGGGTTCGTTATTTGATGAGGGTGCGCCATATCAGCTAGTTGGTGGG

GTAACGGCCTACCAAGGCAATGACGTGTAGCTATGCTGAGAAGTAGAATAGCCACAATGGGACTG

AGACACGGCCCATACTCCTACGGGAGGCAGCAGTAGGGAATTTTTCACAATGAGCGAAAGCTTGA

TGGAGCAATGCCGCGTGAACGATGAAGGTCTTTAAGATTGTAAAGTTCTTTTATTTGGGAAGAATG

ACTTTAGCAGGTAATGGCTAGAGTTTGACTGTACCATTTTGAATAAGTGACGACTAACTATGTGCC

AGCAGTCGCGGTAATACATAGGTCGCAAGCGTTATCCGGATTTATTGGGCGTAAAGCAAGCGCAG

GCGGATTGAAAAGTCTGGTGTTAAAGGCAGCTGCTTAACAGTTGTATGCATTGGAAACTATTAATC

TAGAGTGTGGTAGGGAGTTTTGGAATTTCATGTGGAGCGGTGAAATGCGTAGATATATGAAGGAAC

ACCAGTGGCGAAGGCGAAAACTTAGGCCATTACTGACGCTTAGGCTTGAAAGTGTGGGGAGCAAA

TAGGATTAGATACCCTAGTAGTCCACACCGTAAACGATAGATACTAGCTGTCGGGGCGATCCCCTC

GGTAGTGAAGTTAACACATTAAGTATCTCGCCTGGGTAGTACATTCGCAAGAATGAAACTCAAACG

GAATTGACGGGGACCCGCACAAGTGGTGGAGCATGTTGCTTAATTCGACGGTACACGAAAAACCTT

ACCTAGACTTGACATCCTTGGCAAAGTTATGGAAACATAATGGAGGTTAACCGAGTGACAGGTGGT

GCATGGTTGTCGTCAGCTCGTGTCGTGAGATGTTGGGTTAAGTCCCGCAACGAGCGCAACCCTTAT

CGTTAGTTACATTGTCTAGCGAGACTGCTAATGCAAATTGGAGGAAGGAAGGGATGACGTCAAATC

ATCATGCCCCTTATGTCTAGGGCTGCAAACGTGCTACAATGGCCAATACAAACAGTCGCCAGCTTG

TAAAAGTGAGCAAATCTGTAAAGTTGGTCTCAGTTCGGATTGAGGGCTGCAATTCGTCCTCATGAA

GTCGGAATCACTAGTAATCGCGAATCAGCTATGTCGCGGTGAATACGTTCTCGGGTCTTGTACACA

CCGCCCGTCAAACTATGAAAGCTGGTAATATTTAAAAACGTGTTGCTAACCATTAGGAAGCGCATG

TCAAGGATAGCACCGGTGATTGGAGTTAAGTCGTAACAAGGTACCCCTACGAGAACGTGGGGGTG

GATCACCTCCTTTCTAATGGAG

>CP010544.118311.119846 *Mycoplasma pneumoniae* 85084

AATCTGTCAATTTTTCTGAGAGTTTGATCCTGGCTCAGGATTAACGCTGGCGGCATGCCTAATACAT

GCAAGTCGATCGGAAGTAGTAATACTTTAGAGGCGAACGGGTGAGTAACACGTATCCAATCTACCT

TATAATGGGGGATAACTAGTTGAAAGACTAGCTAATACCGCATAAGAACTTTGGTTCGCATGAATC

AAAGTTGAAAGGACCTGCAAGGGTTCGTTATTTGATGAGGGTGCGCCATATCAGCTAGTTGGTGGG

GTAACGGCCTACCAAGGCAATGACGTGTAGCTATGCTGAGAAGTAGAATAGCCACAATGGGACTG

AGACACGGCCCATACTCCTACGGGAGGCAGCAGTAGGGAATTTTTCACAATGAGCGAAAGCTTGA

TGGAGCAATGCCGCGTGAACGATGAAGGTCTTTAAGATTGTAAAGTTCTTTTATTTGGGAAGAATG

ACTTTAGCAGGTAATGGCTAGAGTTTGACTGTACCATTTTGAATAAGTGACGACTAACTATGTGCC

AGCAGTCGCGGTAATACATAGGTCGCAAGCGTTATCCGGATTTATTGGGCGTAAAGCAAGCGCAG

GCGGATTGAAAAGTCTGGTGTTAAAGGCAGCTGCTTAACAGTTGTATGCATTGGAAACTATTAATC

TAGAGTGTGGTAGGGAGTTTTGGAATTTCATGTGGAGCGGTGAAATGCGTAGATATATGAAGGAAC

ACCAGTGGCGAAGGCGAAAACTTAGGCCATTACTGACGCTTAGGCTTGAAAGTGTGGGGAGCAAA

TAGGATTAGATACCCTAGTAGTCCACACCGTAAACGATAGATACTAGCTGTCGGGGCGATCCCCTC

GGTAGTGAAGTTAACACATTAAGTATCTCGCCTGGGTAGTACATTCGCAAGAATGAAACTCAAACG

GAATTGACGGGGACCCGCACAAGTGGTGGAGCATGTTGCTTAATTCGACGGTACACGAAAAACCTT

ACCTAGACTTGACATCCTTGGCAAAGTTATGGAAACATAATGGAGGTTAACCGAGTGACAGGTGGT

GCATGGTTGTCGTCAGCTCGTGTCGTGAGATGTTGGGTTAAGTCCCGCAACGAGCGCAACCCTTAT

CGTTAGTTACATTGTCTAGCGAGACTGCTAATGCAAATTGGAGGAAGGAAGGGATGACGTCAAATC
ATCATGCCCCTTATGTCTAGGGCTGCAAACGTGCTACAATGGCCAATACAAACAGTCGCCAGCTTG
TAAAAGTGAGCAAATCTGTAAAGTTGGTCTCAGTTCGGATTGAGGGCTGCAATTCGTCCTCATGAA
GTCGGAATCACTAGTAATCGCGAATCAGCTATGTCGCGGTGAATACGTTCTCGGGTCTTGTACACA
CCGCCCGTCAAACTATGAAAGCTGGTAATATTTAAAAACGTGTTGCTAACCATTAGGAAGCGCATG
TCAAGGATAGCACCGGTGATTGGAGTTAAGTCGTAACAAGGTACCCCTACGAGAACGTGGGGGTG
GATCACCTCCTTTCTAATGGAG

> *Mycoplasma pneumoniae* NRBC 14401

GATTAACGCTGGCGGCATGCCTAATACATGCAAGTCGATCGAAAGTAGTAATACTTTAGAGGCGAA
CGGGTGAGTAACACGTATCCAATCTACCTTATAATGGGGGATAACTAGTTGAAAGACTAGCTAATA
CCGCATAAGAACTTTGGTTCGCATGAATCAAAGTTGAAAGGACCTGCAAGGGTTCGTTATTTGATG
AGGGTGCGCCATATCAGCTAGTTGGTGGGGTAACGGCCTACCAAGGCAATGACGTGTAGCTATGCT
GAGAAGTAGAATAGCCACAATGGGACTGAGACACGGCCCATACTCCTACGGGAGGCAGCAGTAGG
GAATTTTTCACAATGAGCGAAAGCTTGATGGAGCAATGCCGCGTGAACGATGAAGGTCTTTAAGAT
TGTAAAGTTCTTTTATTTGGGAAGAATGACTTTAGCAGGTAATGGCTAGAGTTTGACTGTACCATTT
TGAATAAGTGACGACTAACTATGTGCCAGCAGTCGCGGTAATACATAGGTCGCAAGCGTTATCCGG
ATTTATTGGGCGTAAAGCAAGCGCAGGCGGATTGAAAAGTCTGGTGTTAAAGGCAGCTGCTTAACA
GTTGTATGCATTGGAAACTATTAATCTAGAGTGTGGTAGGGAGTTTTGGAATTTCATGTGGAGCGG
TGAAATGCGTAGATATATGAAGGAACACCAGTGGCGAAGGCGAAAACTTAGGCCATTACTGACGC
TTAGGCTTGAAAGTGTGGGGAGCAAATAGGATTAGATACCCTAGTAGTCCACACCGTAAACGATA
GATACTAGCTGTCGGGGCGATCCCCTCGGTAGTGAAGTTAACACATTAAGTATCTCGCCTGGGTAG
TACATTCGCAAGAATGAAACTCAAACGGAATTGACGGGGACCCGCACAAGTGGTGGAGCATGTTG
CTTAATTCGACGGTACACGAAAAACCTTACCTAGACTTGACATCCTTGGCAAAGTTATGGAAACAT
AATGGAGGTTAACCGAGTGACAGGTGGTGCATGGTTGTCGTCAGCTCGTGTCGTGAGATGTTGGGT
TAAGTCCCGCAACGAGCGCAACCCTTATCGTTAGTTACATTGTCTAGCGAGACTGCTAATGCAAAT
TGGAGGAAGGAAGGGATGACGTCAAATCATCATGCCCCTTATGTCTAGGGCTGCAAACGTGCTACA
ATGGCCAATACAAACAGTCGCCAGCTTGTAAAAGTGAGCAAATCTGTAAAGTTGGTCTCAGTTCGG
ATTGAGGGCTGCAATTCGTCCTCATGAAGTCGGAATCACTAGTAATCGCGAATCAGCTATGTCGCG
GTGAATACGTTCTCGGGTCTTGTACACACCGCCCGTCAAACTATGAAAGCTGGTAATATTTAAAAA
CGTGTTGCTAACCATTAGGAAGCGCATGTCAAGGATAGCACCGGTGATTGGAGTTAAG
