# Supplementary material for: Mycoplasma pneumoniae carriage in children with recurrent respiratory tract infections is associated with a less diverse and altered microbiota
Source: eBioMedicine. 2023 Nov 10;98:104868. doi: 10.1016/j.ebiom.2023.104868 (PMC10679896; doi:10.1016/j.ebiom.2023.104868)
Supplement: Study Protocol [file mmc2.pdf]

DIMER-Mycoplasma study (sub-study of the PID study)

DIMER: Deficiency of Immunoglobulin A (IgA) and Microbiome in Respiratory tract infections
PID: Primary Immune Deficiency

Medical ethical approval

The PID study, of which the DIMER-Mycoplasma study is a sub-study, received ethical approval from the Medical Ethical Committee of the Erasmus MC (METC: NL40331.078). All (legal guardians of) subjects signed informed consent and the study was carried out in accordance with the Declaration of Helsinki.

Brief description of DIMER-Mycoplasma sub-study

The DIMER study is a cohort study including children between six weeks and eight years with recurrent respiratory tract infections (rRTIs) and their family members. One of the primary aims is to compare the prevalence of primary antibody deficiencies and *Mycoplasma pneumoniae* carriage in children with rRTIs to controls without infections. In addition, we aim to investigate the mucosal immune system, including differences in mucosal antibody levels and the nasopharyngeal microbiota composition, in children with rRTIs with and without *M. pneumoniae* carriage.

Detailed description of DIMER-Mycoplasma sub-study

Background

rRTIs frequently affect children (1, 2), with around one-third of children with rRTIs also suffering from recurrent lower RTIs. This can be particularly devastating for young children, as alveoli develop up to the age of seven years (3). As a result of rRTIs at a young age, children may experience lung function loss and chronic obstructive pulmonary disease (COPD) later in life (4-6). Following the introduction of pneumococcal conjugate vaccines, *Mycoplasma pneumoniae* has emerged as the most prevalent bacterial cause of community-acquired pneumonia in hospitalized children in Western countries (7, 8). Like other respiratory pathogens, *M. pneumoniae* pneumonia can be preceded by asymptomatic *M. pneumoniae* carriage in the upper respiratory tract (9, 10). The carriage of potential pathogens is influenced by various factors, including the local respiratory microbiota (11). Furthermore, antibody responses are known to be important for immune protection against *M. pneumoniae* (12).

Objectives

1. To provide an overview of the prevalence of *M. pneumoniae* carriage in young children with rRTIs and compare this to their family members without infections.
2. To investigate mucosal and systemic immune factors, including (specific) mucosal antibody levels and the local respiratory microbiota, in relation to *M. pneumoniae* carriage in young children with rRTIs.

Study subjects

In- and exclusion criteria

Children with recurrent respiratory tract infections:

Inclusion criteria:

Children aged six weeks to eight years (until the day they turn eight years old) who are referred to a participating hospital for rRTIs with an indication for immunological screening. rRTIs are defined according to the guideline of the Dutch Section of Pediatric Infectious Diseases & Immunology, and as described by Gruber et al. (13, 14). The choice for immunological screening is based on the physician's professional opinion after the clinical consultation and physical examination. Furthermore, children with an already known IgA deficiency (2SD below age-appropriate reference values (see appendix 1)) diagnosed within the year before inclusion, are also included.

Exclusion criteria:

1) Primary immunodeficiency for which intravenous immunoglobulin (IVIG) treatment has started,
2) Secondary immunodeficiencies (e.g. HIV, chemotherapy, transplantation),
3) Major congenital anomalies (eponymous syndromes, chromosomal abnormalities, cleft palate, renal or hepatic insufficiency, known glucose-6-phosphate deficiency, cystic fibrosis, primary ciliary dyskinesia or acute porphyria),
4) Antibiotic use (prophylactic or course) four weeks prior to inclusion (if this is the only exclusion criteria, a child can be included but a mucosal sample should be collected four weeks after the last antibiotic course or prophylactic treatment). Furthermore, subjects with azithromycin prophylaxis three months prior to sampling are also excluded, since this could influence *M. pneumoniae* carriage,
5) Both parents below the age of 18 years,
6) Language barrier that prevents a proper informed consent procedure,
7) No informed consent.

Controls:

Inclusion criteria:

Family members of children with rRTIs included in the DIMER study, living in the same household (for most of the time). A family member does not need to be a biological family member if they live in the same household as the patient with rRTIs. Multiple controls can be included per child with rRTIs.

Exclusion criteria:

1) Known immunodeficiencies (except IgA deficiency),
2) Recurrent respiratory tract infections,
3) Secondary immunodeficiencies,
4) Major congenital anomalies,
5) Antibiotic use (prophylactic or course) four weeks prior to inclusion (if this is the only exclusion criteria, a family member can be included but a mucosal sample should be collected four weeks after the last antibiotic course or prophylactic treatment).

Furthermore, subjects with azithromycin prophylaxis three months prior to sampling are also excluded, since this could influence *M. pneumoniae* carriage,

6) In the case of a sibling below the age of 16 years: both parents below the age of 18 years,
7) Language barrier that prevents a proper informed consent procedure,
8) No informed consent.

Workflow identifying potential study subjects and providing study information

Children with recurrent respiratory tract infections:
New patients that may fulfill the inclusion criteria of the study will be identified from the outpatient clinic by the treating physician and/or a member of the study team. Caretakers of patients will be called one to two weeks in advance and receive oral and written information from the study team.
During the outpatient clinic visit, the treating physician will decide whether an immunological screening is appropriate and inform the study team. If a patient is indeed a candidate (after checking the in- and exclusion criteria), the informed consent will be signed by caretakers and a member of the study team prior to a blood draw, mucosal sample collection, and filling in the inclusion questionnaire. A mucosal sample will only be collected if there is no antibiotic use in the previous four weeks. Later sampling (after a period of four weeks without antibiotic use) can occur during a home visit if no further hospital visits are planned.

Controls (family members of children with rRTIs)
After the inclusion of a subject with rRTIs, family members will be asked to participate in the study if they fulfill the in- and exclusion criteria. Again, information about the study is supplied beforehand and the informed consent procedure will take place prior to sample and data collection as described above. For young siblings, a blood draw can also take place via a finger prick if this is the caretakers' wish.

Sampling
<u>Collection of samples</u>
Children with recurrent respiratory tract infections:
Mucosal samples: nasopharyngeal swab, saliva, and feces.
Blood samples: serum, plasma, EDTA, and PBMC's. Blood will only be collected from children with rRTIs when there is a clinical indication for blood drawing.

Family members
Mucosal samples: nasopharyngeal swab, saliva, and feces.
Blood samples: serum, plasma, EDTA, and PBMC's. Blood collection will only take place once.

<u>Timing of sampling</u>

- In subjects <u>without</u> antibiotic use in the previous four weeks: all samples, including blood, will be collected at inclusion.
- In subjects <u>with</u> antibiotic use in the previous four weeks and in whom a pneumococcal vaccine response will be measured in 4-6 weeks: all samples, including blood, will be collected when pneumococcal response is measured.

- In subjects with antibiotic use in the previous four weeks, in whom no pneumococcal vaccine response will be measured in 4-6 weeks: blood samples will be collected at inclusion and a mucosal sample will be collected at a later time point (next outpatient appointment or home visit) when the subject has no antibiotic use in the previous four weeks.

Sampling procedures and analyses

All samples will be collected by trained study personnel and placed on dry ice immediately after collection. Nasopharyngeal swabs will be taken in the nasopharyngeal cavity and rotated clockwise three times and counterclockwise three times, before being stored in RNA protect medium (Qiagen). Saliva will be collected by placing a saliva collection swab (Oracle) in the mouth of the subject. Saliva will be divided over three media: an EDTA (BD), glycerol and RNA protect (Qiagen). If contamination takes place (e.g. if the swab touches a cheek or a hand) a new sample will be taken. Fecal collection tubes are provided to all (caretakers of) subjects at inclusion and (caretakers of) subjects are requested to collect feces within 48 hours and store these in the freezer until the next hospital visit or until a member of the study team comes to collect it during a house visit. All samples will be transported on dry ice and stored at -80 degrees Celsius until further processing.

Nasopharyngeal swabs will be used for respiratory microbiota measurement with 16S-rRNA-sequencing and for the detection of *M. pneumoniae* and viruses (viral panel of 12 respiratory viruses) with qPCR. Viral qPCR was only performed in a subset of children of whom a nasopharyngeal swab was collected at the start the winter season of 2016 till 2019 and if parents were willing to fill in a cellphone application for four consecutive winter months to register daily RTI symptoms as part of a separate study (prospective RTI symptom registration study).

In addition, total IgA and *M. pneumoniae-specific* IgA will be measured in the nasopharyngeal swab using ELISA.

Serum will be used to measure serum IgA, IgG, IgG subclasses, and IgM levels.

Clinical data

At inclusion, (caretakers of) subjects will be asked to fill in a questionnaire on their medical history, underlying conditions, and medication use. Furthermore, a longer questionnaire on all medical history, allergies, and asthma will be sent to all caretakers of children with rRTIs. At inclusion, length and weight will be measured and a physical examination will take place. Lab results from the immunological screening will be extracted from the electronic medical file. RTI symptoms at the time of sampling will be registered.

Infection symptoms at the time of sampling (categorized):

1) No symptoms
2) Mild RTI (only 1 RTI symptom (see below) or >1 RTI symptom for 1 day only)
3) Moderate RTI **without antibiotic use**: $\geq$2 RTI symptoms for $\geq$2 days without antibiotic use
4) Moderate RTI **with antibiotic use**: $\geq$2 RTI symptoms for $\geq$2 days with antibiotic use

5) Severe RTI: hospital admission because of RTI

RTI symptoms:

a. fever > 38.0 degrees
b. rhinitis
c. otitis
d. pharyngitis/sore throat
e. hoarseness
f. coughing
g. wheezing
h. dyspnea
i. agitation/crying

Data storage
All patients will receive a study pseudo-ID number through a certified ID generator. Data will be stored in a certified electronic research database (Research Online) and all members of the study team will follow a course prior to using this database. Missing data will be tried to be retrieved through calling (caretakers of) subjects. If missing data cannot be retrieved, it will be filled in as missing, where possible accompanied with the reason why the data is missing.

Statistical analyses
All clinical and laboratory data will be combined in a single dataset. Microbiota data measured with 16S-rRNA sequencing will be filtered and annotated through standardized methods from our study group and stored as a separate file. All analyses will be conducted using R and R Studio.

Baseline characteristics will be compared with Chi-square/Fishers exact test and Mann-Whitney U test. Correlations will be identified using Pearson's/Spearman's correlation test. Where appropriate, analyses will be corrected for age as a possible confounding variable. *M. pneumoniae* carrier status will be compared with univariable and multivariable logistic regression analysis (using the Akaike Information Criterion comparing the fit of subsequent models).
For the microbiota data, alpha diversity will be measured using the Shannon index and beta diversity will be based on the Bray-Curtis dissimilarity matrix. All further microbiota analyses will be based on total sum scaled (relative) abundances.

References

1. Schaad U, Esposito S, Razi C. Diagnosis and Management of Recurrent Respiratory Tract Infections in Children: A Practical Guide. Arch Pediatr Infect Dis. 2016;4(1).
2. Pasternak G, Lewandowicz-Uszynska A, Krolak-Olejnik B. Recurrent respiratory tract infections in children. Pol Merkur Lekarski. 2020;49(286):260-6.
3. Shi W, Bellusci S, Warburton D. Lung development and adult lung diseases. Chest. 2007;132(2):651-6.

4. Yang IA, Jenkins CR, Salvi SS. Chronic obstructive pulmonary disease in never-smokers: risk factors, pathogenesis, and implications for prevention and treatment. Lancet Respir Med. 2022;10(5):497-511.
5. Ozkan H, Atlihan F, Genel F, Targan S, Gunvar T. IgA and/or IgG subclass deficiency in children with recurrent respiratory infections and its relationship with chronic pulmonary damage. J Investig Allergol Clin Immunol. 2005;15(1):69-74.
6. Svanes C, Sunyer J, Plana E, Dharmage S, Heinrich J, Jarvis D, et al. Early life origins of chronic obstructive pulmonary disease. Thorax. 2010;65(1):14-20.
7. Meyer Sauteur PM, Krautter S, Ambroggio L, Seiler M, Paioni P, Relly C, et al. Improved Diagnostics Help to Identify Clinical Features and Biomarkers That Predict Mycoplasma pneumoniae Community-acquired Pneumonia in Children. Clin Infect Dis. 2020;71(7):1645-54.
8. Jain S, Williams DJ, Arnold SR, Ampofo K, Bramley AM, Reed C, et al. Community-acquired pneumonia requiring hospitalization among U.S. children. N Engl J Med. 2015;372(9):835-45.
9. Bogaert D, van Belkum A, Sluijter M, Luijendijk A, de Groot R, Rumke HC, et al. Colonisation by Streptococcus pneumoniae and Staphylococcus aureus in healthy children. Lancet. 2004;363(9424):1871-2.
10. Spuesens EB, Fraaij PL, Visser EG, Hoogenboezem T, Hop WC, van Adrichem LN, et al. Carriage of Mycoplasma pneumoniae in the upper respiratory tract of symptomatic and asymptomatic children: an observational study. PLoS Med. 2013;10(5):e1001444.
11. de Steenhuijsen Piters WAA, Jochems SP, Mitsi E, Rylance J, Pojar S, Nikolaou E, et al. Interaction between the nasal microbiota and S. pneumoniae in the context of live-attenuated influenza vaccine. Nat Commun. 2019;10(1):2981.
12. de Groot RCA, Cristina Estevao S, Meyer Sauteur PM, Perkasa A, Hoogenboezem T, Spuesens EBM, et al. Mycoplasma pneumoniae carriage evades induction of protective mucosal antibodies. Eur Respir J. 2021.
13. Gruber C, Keil T, Kulig M, Roll S, Wahn U, Wahn V, et al. History of respiratory infections in the first 12 yr among children from a birth cohort. Pediatr Allergy Immunol. 2008;19(6):505-12.
14. Driessen GJA. Evidence-based richtlijn diagnostiek naar onderliggende aandoeningen bij kinderen met recidiverende luchtweginfecties: Erasmus MC, Sophia Kinderziekenhuis, subafdeling kinderinfectieziekten-immunologie; 2016 [Available from: https://www.kinderinfectieziekten.nl/wp-content/uploads/2016/09/12-07-2016-Diagnostiek-recidiverende-luchtweginfecties.pdf.

Appendix 1

Definition of antibody deficiencies: An IgA, IgG (subclass) or IgM deficiency was defined as having an IgA or IgG (subclass) level below minus two standard deviation (-2SD) of age-specific reference values.

The following reference values, as used in Dutch clinical practice, were applied:

Reference values for IgA and IgG: range from -2SD to +2SD for different ages.

Age	IgA (g/l)	IgG (total) (g/l)	IgM (g/l)
0–2 weeks	<0,16	6,5-12,6	0,03-0,24
0,5–4 months	0,06-0,57	2,6-7,8	0,10-0,55
4–6 months	0,08-0,90	2,2-11,3	0,07-0,65
6–12 months	0,16-0,98	2,6-15,2	0,17-1,20
1–2 years	0,19-1,10	2,6-13,9	0,10-0,87
2–3 years	0,19-2,30	4,3-13,0	0,21-0,87
3–6 years	0,55-2,20	5,2-13,4	0,24-1,80
6–9 years	0,54-2,50	5,2-14,3	0,28-1,90

Reference values for IgG subclasses: range from -2SD to +2SD for different ages.

Age	IgG1 (g/l)	IgG2 (g/l)	IgG3 (g/l)	IgG4 (g/l)
0-1 months	2.4-10.6	0.87-4.10	0.14-0.55	0.04-0.56
1-4 months	1.8-6.7	0.38-2.10	0.14-0.70	0.02-0.36
4-6 months	1.8-7.0	0.34-2.10	0.15-0.80	0.02-0.23
0,5-1 years	2.0-7.7	0.34-2.30	0.15-0.97	0.01-0.43
1-1.5 years	2.5-8.2	0.38-2.40	0.15-1.07	0.01-0.62
1.5-2 years	2.9-8.5	0.45-2.60	0.15-1.13	0.01-0.79
2-3 years	3.2-9.0	0.52-2.80	0.14-1.20	0.01-1.06
3-4 years	3.5-9.4	0.63-3.00	0.13-1.26	0.02-1.27
4-6 years	3.7-10.0	0.72-3.40	0.13-1.33	0.02-1.58
6-9 years	4.0-10.8	0.85-4.10	0.13-1.42	0.02-1.89
